# Metabolic Consequences of Developmental Exposure to Polystyrene Nanoplastics, the Flame Retardant BDE-47 and Their Combination in Zebrafish

**DOI:** 10.3389/fphar.2022.822111

**Published:** 2022-02-16

**Authors:** Raphaël Chackal, Tyler Eng, Emille M. Rodrigues, Sara Matthews, Florence Pagé-Lariviére, Stephanie Avery-Gomm, Elvis Genbo Xu, Nathalie Tufenkji, Eva Hemmer, Jan A. Mennigen

**Affiliations:** ^1^ Department of Biology, University of Ottawa, Ottawa, ON, Canada; ^2^ Department of Chemistry and Biomolecular Sciences, University of Ottawa, Ottawa, ON, Canada; ^3^ Department of Chemical Engineering, McGill University, Montréal, QC, Canada; ^4^ National Wildlife Research Center, Environment and Climate Change Canada, Ottawa, ON, Canada; ^5^ Department of Biology, University of Southern Denmark, Odense, Denmark

**Keywords:** brominated flame retardants, legacy contaminants, nanoplastics, emerging contaminant, energy metabolism, gene expression, cumulative effects

## Abstract

Single-use plastic production is higher now than ever before. Much of this plastic is released into aquatic environments, where it is eventually weathered into smaller nanoscale plastics. In addition to potential direct biological effects, nanoplastics may also modulate the biological effects of hydrophobic persistent organic legacy contaminants (POPs) that absorb to their surfaces. In this study, we test the hypothesis that developmental exposure (0–7 dpf) of zebrafish to the emerging contaminant polystyrene (PS) nanoplastics (⌀100 nm; 2.5 or 25 ppb), or to environmental levels of the legacy contaminant and flame retardant 2,2′,4,4′-Tetrabromodiphenyl ether (BDE-47; 10 ppt), disrupt organismal energy metabolism. We also test the hypothesis that co-exposure leads to increased metabolic disruption. The uptake of nanoplastics in developing zebrafish was validated using fluorescence microscopy. To address metabolic consequences at the organismal and molecular level, metabolic phenotyping assays and metabolic gene expression analysis were used. Both PS and BDE-47 affected organismal metabolism alone and in combination. Individually, PS and BDE-47 exposure increased feeding and oxygen consumption rates. PS exposure also elicited complex effects on locomotor behaviour with increased long-distance and decreased short-distance movements. Co-exposure of PS and BDE-47 significantly increased feeding and oxygen consumption rates compared to control and individual compounds alone, suggesting additive or synergistic effects on energy balance, which was further supported by reduced neutral lipid reserves. Conversely, molecular gene expression data pointed to a negative interaction, as co-exposure of high PS generally abolished the induction of gene expression in response to BDE-47. Our results demonstrate that co-exposure to emerging nanoplastic contaminants and legacy contaminants results in cumulative metabolic disruption in early development in a fish model relevant to eco- and human toxicology.

## 1 Introduction

As the rapidly increasing production of plastic overwhelms the world’s ability to efficiently manage its disposal, plastic pollution has quickly become one of the most pressing environmental issues. Since 2013, the global annual production of plastic has exceeded 300 million tonnes (Mt) with rates reaching as high as 368 Mt in 2019 (www.plasticseurope.org). Due to their durability, low recycling rates and poor waste management, a significant portion of the plastic produced worldwide enters and persists in marine and, to a lesser degree, freshwater aquatic ecosystems (Barnes et al., 2009; Lebreton et al., 2017). It has been estimated that between 4.8 and 12.7 MT of plastic infiltrate oceans every year from coastal populations worldwide (Lebreton et al., 2017). In freshwater samples from the Western Lake Superior, estuary and harbour samples averaged 54,000 plastic particles/km^2^, followed by open water samples averaging 38,000 particles/km^2^ and then nearshore samples averaging 28,000 particles/km^2^ (Hendrickson et al., 2018).

Once released into aquatic systems, plastics are exposed to varying weathering conditions; factors such as water turbulence, erosion and intense sunlight exposure degrade large plastic fragments into increasingly smaller particles on the micro- and nanosized scales (Ter Halle et al., 2017). Nanoplastics are small plastic particles that exhibit characteristics distinct from microplastics (Gigault et al., 2021). Polystyrene (PS) plastics are among the most abundant plastics detected in aquatic ecosystems. A study of microplastic pollution in the Bohai Sea, a model chosen because it is almost entirely enclosed by land and thus exhibits limited self-cleaning abilities, found PS to be the third most abundant plastic particle after polyethene and polypropylene (Zhang et al., 2017). In another study, water samples obtained from the coastlines of the Canterbury region of New Zealand showed that PS made up 55% of all particles identified (Clunies-Ross et al., 2016).

While the hazards associated with macro- and microplastics to aquatic organisms are relatively well characterized, the bioaccumulation and toxicity of nanoplastics are only beginning to be considered even though they could potentially be more hazardous (Koelmans et al., 2015b; Mitrano et al., 2021). Thus far, several studies have shown that nanoplastics can transport through the food web (Mattsson et al., 2017), translocate between organs in the body (Farrell and Nelson, 2013) and transfer from mothers to offspring (Pitt et al., 2018). The potential of PS nanoplastic particles to bioconcentrate through dietary exposure was established in a study exploring the accumulation of fluorescent particles in the food chain (Chae et al., 2018). The study involved phytoplankton *Chlamydomonas reinhardtii* (a producer), zooplankton *Daphnia magna* (primary consumer), *Oryzias sinensis* (secondary consumer) and dark chub *Zacco temmincki* (tertiary consumer). Although only the phytoplankton was exposed to 50 mg/L of PS nanoplastics, microscopic observations demonstrated that PS nanoplastic particles were present in digestive organs of the primary, secondary, and tertiary consumers. The accumulated nanoplastic further penetrated fish embryos and was detected in their yolk sac (Chae et al., 2018). The concern for plastic particles to reach higher trophic levels extends to humans (Farrell and Nelson, 2013; Watts et al., 2014). A study examining human colectomy specimens in long-time coastal residents found on average 28.1 particles/g tissue (Ibrahim et al., 2021). Microplastics were detected in human stool in the order of 2 microplastic particles/g (Schwabl et al., 2019). A recent study estimated that globally, on average, humans may ingest 0.1–5 g of microplastics weekly through various exposure pathways (Senathirajah et al., 2021). While less information on human tissue levels is currently available for nanoplastics, these studies reveal that nanoplastics may, in addition to ecotoxicological concerns, also pose a direct risk to human health (Revel et al., 2018). In addition to the possibility to elicit biological effects independently, nanoplastics such as PS have been shown to interact with persistent organic pollutants (POP) (Lee et al., 2014) like BDE-47 (Xu et al., 2019; Sun et al., 2021). Such interactions have raised the question of potential vector or sequestering effects of nanoplastic with regard to POPs, which may promote bioaccumulation of POPs on the one hand or limit their bioavailability on the other. Thus, the investigation of the combined biological effects of nanoplastic and POP mixtures in freshwater fish is warranted.

2,2′,4,4′-Tetrabromodiphenyl ether (BDE-47), a Polybrominated Diphenyl Ether (PBDE) is one example of a legacy POP with an absorption capacity that is particularly high for PS compared to other microplastics (Xu et al., 2019). Historically used in PBDE mixtures as a flame retardant in materials including polyurethane and firefighting foam, BDE-47 has been voluntarily phased out in Europe since 2003 and in the U.S. since 2004. However, due to its chemical inertness and physicochemical properties including a high octanol-water partition coefficient (*K*_OW_) of 6.57, significant amounts of the world’s water, terrestrial land, and most animals and humans contain traces of BDE-47: In United Kingdom and United States freshwater lakes, average ΣBDE water sample concentrations largely dominated by BDE-47 were reported in the pg/L range (Streets et al., 2006; Yang et al., 2014). BDE-47 is also the predominant BDE species in Great Lake top predator fish, which after peaking at ∼150 ng/g in 2000 have declined to concentrations <50 ng/g in 2015 (Zhou et al., 2019). BDE-47 is also of concern to humans, who are exposed via water, food and atmosphere (Webster et al., 2005). Concentrations of BDE-47 in humans in North America are approximately 35 ng/g of lipid (Hites, 2004). In addition to its propensity to bioconcentrate and biomagnify (Kamel et al., 2012), BDE-47 is transferred to offspring *via* egg deposition in fish (Wen et al., 2015) and placental transfer and lactation in mammals (Mazdai et al., 2003; Frederiksen et al., 2010; Koenig et al., 2012). These data highlight concerns regarding consequences of developmental BDE-47 exposure in multiple species (Mohammed, 2013).

Among the biological effects of both PS nanoplastics and BDE-47 exposure, evidence points to energy metabolism as a common endpoint. In fish, PS nanoplastics have been reported to affect morphometric parameters such as body weight, behaviours relevant to organismal energy balance such as feed-intake, interfere with intestinal nutrient absorption (Jovanović, 2017), dysregulate glucose and lipid metabolism (Cedervall et al., 2012; Brun et al., 2019), and reduce mitochondrial function (Trevisan et al., 2019). Similar effects on energy metabolism are emerging from studies in mammalian systems (Yee et al., 2021). In fish, BDE-47 has been demonstrated to reduce body weight (Torres et al., 2013), induce motor deficits (Chen et al., 2012) and disrupt mitochondrial biogenesis, dynamics and function (Zhuang et al., 2020). Effects of BDE-47 on energy metabolism are corroborated by epidemiological and rodent model studies, which support a role for developmental BDE-47 exposure in the etiology of diabetes and associated perturbations in glucose and lipid metabolism (McIntyre et al., 2015; Zhang et al., 2016; Wang D et al., 2018).

Taking advantage of the high-throughput zebrafish model relevant to eco- and human toxicology (Dai et al., 2014; Garcia et al., 2016; Bambino and Chu, 2017), early development (Kimmel et al., 1995) and metabolic disease (Seth et al., 2013; Benchoula et al., 2019), we here test the hypothesis that (I) acute developmental exposure to PS or environmental levels of BDE-47 alone disrupts larval energy metabolism and (II) their mixture will exacerbate metabolic disruption.

## 2 Materials and Methods

### 2.1 Validation of PS Nanoplastic Uptake by Fluorescence Microscopy

To validate bioaccumulation of PS nanoparticles in wildtype TU strain zebrafish, eleutheroembryos were either left unexposed (*n* = 3) or exposed to Firefli Fluorescent Green labelled 100 nm ⌀ PS nanoplastics (Catalogue # G100, Thermo-Fisher Scientific, Ottawa, ON, Canada) at concentrations of 2.5 (*n* = 3) and 25 ppm (*n* = 3) in a glass Petri dish containing 25 ml of RO salt-dosed (Instant Ocean, PetSmart, Ottawa, ON, Canada) University of Ottawa Aquatics Facility system water (pH = 7.4, conductivity = μS) maintained at 28°C and from 0–7 days post fertilization (dpf) under a 12:12 L:D photoperiod. Prior to experimental use, particles were dialyzed to remove free dye and additives from the PS nanoparticle formulation as previously described (Pikuda et al., 2019; Xu et al., 2019). Zebrafish were euthanized with tricaine at 7 dpf, fixed in 4% PFA at 4°C o/n and then transferred to 80% ethanol. Whole zebrafish were placed on a glass slide and imaged using a custom-built microscope (IMA Upconversion by PhotonEtc, Montreal, QC, Canada) equipped with an inverted optical microscope (Nikon Eclipse Ti-U), a broadband camera for color imaging, a Princeton Instruments ProEM EMCCD camera for detection of visible emission, a Nikon halogen lamp (IntensiLight 100 W) with a single band FITC filter cube for 490 nm light excitation and 509 nm emission, collected epifluorescently, to screen for PS nanoplastic-emitted fluorescence at the University of Ottawa. To assess tissue-specific PS uptake, adult zebrafish were exposed to fluorescently labelled PS for a period of 4 days in a separate experiment, and tissues processed and analyzed at the Advanced Bioimaging Facility at McGill University as described in Supplementary Figure S1.

### 2.2 Developmental Exposure of Zebrafish (eleuthero)embryos to PS, BDE-47 and Their Combination

**FIGURE 1:**
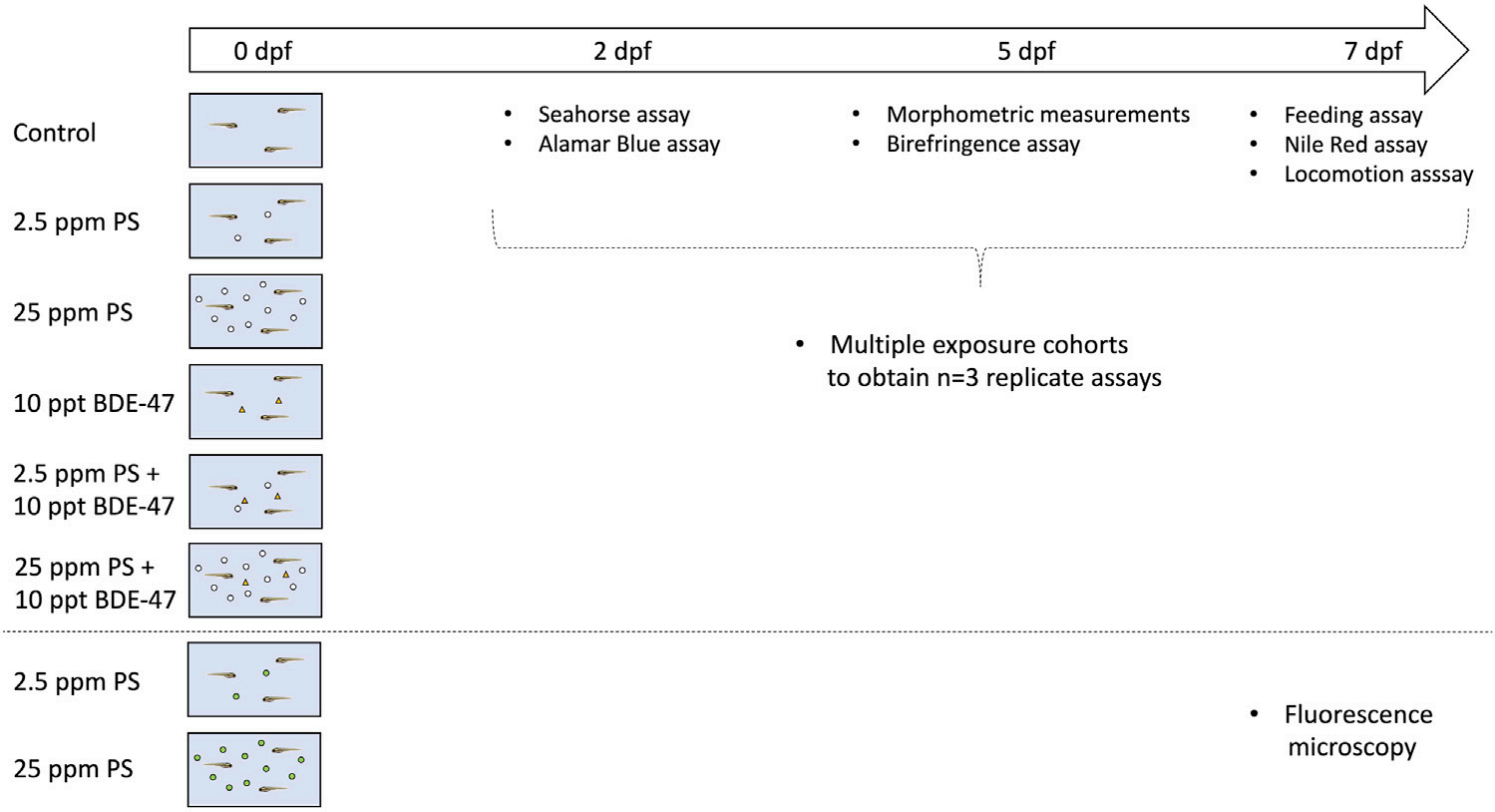
Schematic representation of the experimental design.

To assess metabolic consequences of developmental exposure to PS nanoplastics, BDE-47 and their combination, wildtype TU embryos from multiple breeding pairs were pooled and subsequently randomly divided into 6 treatment groups (*n* = 50 embryos/Petri dish) and exposed to prepared stocks of DMSO vehicle control (<0.001% v/v), dialyzed (Xu et al., 2019), unlabelled 100 nm ⌀ PS nanoplastics (Catalogue # 5010A, Thermo-Fisher Scientific) at 2.5 (low) or 25 ppm (high), 10 ppt analytical grade >97% purity BDE-47 (Catalogue # 91,834 Sigma-Aldrich, Oakville, ON Canada), or their combinations from 0–7 dpf under 12:12 L:D photoperiod in a static, single pulse exposure (Figure 1). All groups were exposed in glass Petri dishes filled with 25 ml of University of Ottawa Aquatic Facility RO-distilled and salt-dosed 28°C system water. In absence of available direct measurements of nanoplastic concentration in natural aquatic environments (Koelmans et al., 2015a), nanoplastic concentrations were based on reported freshwater microplastic ranges (Lu et al., 2021). Multiple cohorts were exposed to obtain necessary sample numbers for three replicate assays for each organismal endpoint measured during the exposure period (Figure 1). Sample sizes were determined a priori based on previous studies reporting metabolic disruption following developmental contaminant exposures in zebrafish larvae in our lab (Tu et al., 2019; Martínez et al., 2020). To avoid contamination or contaminant transfer between cohorts, a three-step treatment process was used. Following manual cleaning with soap and deionized water, the equipment was dried with paper towels and rinsed with hexane. Finally, dishes were rinsed with acetone, dried and cleaned with molecular grade distilled water. All experimental protocols were approved by the University of Ottawa’s Animal Care and Veterinary Service (BL-2786). While following recommended parameter guidelines (water quality, photoperiod, temperature) specified in OECD guidelines for test number 210—Fish, Early-life stage Toxicity test (https://doi.org/10.1787/9789264203785-en), our experimental design deviates from the recommended exposure duration for zebrafish. Thus, while start of exposure occurs prior to blastodisc cleavage, our experimental duration (0-7 dpf) is, while in line with previous contaminant exposures preceding beginning sexual differentiation (Tu et al., 2019), shorter than the recommended zebrafish exposure duration 0–30 dpf.

### 2.3 Assessment of the Organismal-Level Metabolic Phenotype

#### 2.3.1 Morphometric Parameters

Body length and lateral surface area of eleutheroembryos (5 dpf) were quantified by an investigator blind to the treatment groups on ImageJ using images captured with a stereomicroscope (Nikon SMZ 1500) and the Lumenera Infinity 2 camera and Infinity Capture software (Teledyne Lumenera, Ottawa, ON, Canada) as previously described (Martínez et al., 2020). A subset of the same specimens was subsequently used to assess myofilament organization using a birefringence assay as previously described (Berger et al., 2012; Smith et al., 2013; Martínez et al., 2020). Briefly, following bright-field microscopy imaging, a subsequent picture of the eleutheroembryos was taken immediately afterward using two perpendicularly placed 77 mm circular polarizing lenses (MC CPL 77 mm, AmazonBasics, Seattle, United States) to visualize and image birefringence created from the diffraction of polarised light through the pseudo-crystalline array of the muscle sarcomeres. Care was taken to maintain eleutheroembryo position between images. Birefringence intensity was analyzed using ImageJ and normalized to the whole-body area of the larvae. A total of three cohorts were analyzed reaching a total *n* = 20–23 per treatment group for length and surface area measurements and a subset of *n* = 12–19 per treatment group for birefringence assay measurements. The difference in sample size in the birefringence assay is due to removal of larvae imaged in curved positioning prior to analysis, as these result in strongly reduced birefringence readings.

#### 2.3.2 Feeding Behaviour

Food consumption was quantified by feeding zebrafish (7 dpf) with food labelled with a lipophilic fluorescent dye as described previously (Tu et al., 2019; Martínez et al., 2020). Briefly, 30 μL of 4-Di-10-ASP (4-(4-(Didecylamino)styryl)-N-Methylpyridinium iodide) (Thermo-Fisher Scientific) were dissolved in 1.47 ml of analytical grade acetone and mixed with 300 mg of G75 zebrafish feed (Skretting, Stavanger, Norway). The feed was allowed to saturate, covered by tinfoil and then dried under the fumehood in a glass Petri dish for 48 h at RT. Each group of treated zebrafish was fed with excess labelled food and allowed to feed for 1 h in a dark incubator at 27°C. One control group received unlabelled food under the same conditions to assess background fluorescence. After 1 h, fish were anesthetized with tricaine to suspend feeding. An *n* = 9 larvae were collected and pooled as replicate in a microcentrifuge tube, and *n* = 12 pooled replicates were collected per treatment group. Using a micropipette, the water was carefully removed, and larvae were washed repeatedly and finally resuspended in 300 μL of molecular biology grade water. Each pooled replicate was then homogenized using a Pellet Pestle™ homogenizer (Thermo-Fisher Scientific). 250 μL of homogenate was then placed into individual wells of a 96-well Nunc™ Microplate™ (Thermo-Fisher, Scientific) and plates analyzed for fluorescence emission using the SpectraMax Gemini fluorometer (Molecular Devices, Sunnyvale, CA, United States). The excitation wavelength used was 485 nm and the measured emission wavelength was 530 nm. The average background fluorescence values were subtracted from measured values and obtained measurements normalized to average control group values to depict fold-change. A total of three cohorts were analyzed reaching a combined sample size of *n* = 12 per treatment group.

#### 2.3.3 Neutral Lipid Storage

Neutral lipid deposition was measured in 7 dpf larvae using Nile Red (9-Diethylamino-5H-benzo [alpha]phenoxazine-5-one, Sigma-Aldrich), a lipophilic fluorescent stain (Jones et al., 2008; Minchin and Rawls, 2017). Nile red stock was diluted in analytical grade acetone to a concentration of 500 μg/ml. This was then diluted 1:100 in system water and larvae were maintained in 10 ml of this solution for 30 min in the dark at 27°C. Larvae were then rinsed twice in system water and anesthetized with tricaine. Each larva was imaged under a fluorescent stereomicroscope with a super high-pressure mercury lamp (Nikon SMZ 1500). Images were captured with a Lumenera Infinity 2 camera using a Texas Red filter and processed using the Infinity Capture software (Teledyne Lumenera, Ottawa, ON, Canada). All images were taken at the same exposure settings and magnification. Fluorescence was quantified using ImageJ and Nile red stains were normalized to the lateral body area of the fish. A total of three cohorts were analyzed reaching a combined sample size of *n* = 18–22 per treatment group. All images were renamed prior to analysis to assure the experimenter was blind to specific treatment groups during analysis.

#### 2.3.4 Oxidative Metabolism

##### 2.3.4.1 Oxygen Consumption Rate Measurement

The baseline oxygen consumption rate was assessed in 2 dpf eleutheroembryos using a Seahorse Xf24 Analyzer (Agilent, Mississauga, ON, Canada) as previously described (Tu et al., 2019). Briefly, eleutheroembryos were rinsed in Aquatic Facility system water and individually transferred into a 24-well Islet capture plate (#101122–100, Agilent) in 500 μL Aquatic Facility system water using a micropipette. Each well was then sealed using a mesh. Cartridges were prepared 24 h in advance and allowed to equilibrate with calibration solution in a 27°C incubator o/n. A total of three cohorts were analyzed reaching a combined sample size of *n* = 7-9 per treatment group.

##### 2.3.4.2 Oxidative Metabolism-Dependent Energy Expenditure Assay

Cumulative oxygen metabolism-dependent energy expenditure was assessed over a 24 h in 2 dpf eleutheroembryos using the Alamar Blue assay, which is dependent on a NADH-, NADPH-based reduction of Alamar Blue (resazurin) and has been described as proxy to quantify zebrafish oxidative metabolism (Renquist et al., 2013; Williams and Renquist, 2016; Reid et al., 2018). Briefly, eleutheroemberyos from each treatment group were transferred to a solution made up of 0.3 ml Alamarblue Cell Viability Reagent (Thermo-Fisher Scientific), 3.0 ml of 4 mM NaHCO_3_ (aq), 0.03 ml DMSO, and 26.67 ml of system water. Individual eleutheroembryos were then captured in 200 μL of this solution using a micropipette and dispensed into a 96-well plate. The plate was incubated at 27°C in the dark incubator until reading. Two blank wells not containing eleutheroembryos were also prepared for each group and used to acquire the background readings for a 24 h incubation of solution alone. Each plate was then placed in a fluorometer (Molecular Devices) to measure NADH/NADPH emission at 590 nm following excitation at 530 nm. Readings were normalized by subtraction of background readings and obtained values normalized to group values to depict fold-change. A total of three cohorts were analyzed reaching a combined sample size of *n* = 33–47 per treatment group.

#### 2.3.5 Light-Dark Locomotion Assay

The locomotion behavior of 7 dpf zebrafish larvae was assessed at baseline and under different lighting conditions. To achieve this, larvae from each group were placed into individual wells of a 96-well plate with 250 μL of Aquatic Facility system water. The plate was then placed into the ZebraBox Larvae and Embryos Monitor (ViewPoint Behavior Technology, Montréal, QC, Canada) where the larvae were allowed to acclimatize to the full intensity light for 30 min to reduce sampling stress. The larvae were then exposed to instantaneous 100% light/dark intensity changes with the following pattern (times in min, L, light, D, darkness): 20L-5D-5L-5D-5L-5D-5L. The Viewpoint Zebralab v3 quantization software was used to track the individual’s movements and to perform the automated behavioral analysis to obtain the movement time and distance of a given larvae within three defined movement ranges [inactivity (<3 mm/s), short-(3–6 mm/s), and long- (6 mm/s) movements] analyzed for each minute. Speed was calculated by dividing distance by time values. Data were analyzed separately for baseline, light and dark conditions.

### 2.4 Gene Expression

Samples were homogenized using a sonicator and total RNA extracted using the Trizol method. Total RNA purity and quantity were determined using a Nanodrop ™ (Thermo-Fisher Scientific) as previously described (Tu et al., 2019; Martínez et al., 2020). Using an input of 1 μg RNA, cDNA was then generated using Superscript II kit (Thermo-Fisher Scientific) according to the manufacturer’s instructions. Controls omitting template (no template control) and reverse transcriptase (no RT control) were included in subsequent *realtime* RT-PCR assays using SSo Advanced Universal SYBR Green reagents (Bio-Rad, Montréal, QC, Canada). Briefly, relative gene expression was quantified using a two-step protocol on a CFX96 machine (Bio-Rad). Using pooled cDNA, 1:2 serial dilutions were pipetted to generate standard curves which were run in duplicates along with samples for each specific run. The individual reaction volume was 20 μL consisting of 10 μL 2x SSo Advanced Universal SYBR Green Master Mix (BioRad), 1 μL of 10 μM forward and reverse primers and 1 μL of cDNA template. Primer sequences, gene accession numbers and specific annealing temperatures (Tm) are reported in Table 1. For all gene expression assays, efficiencies between 90 and 100% and *R* ^2^ values exceeding 0.98 were considered acceptable. Reactions were monitored for single product by generating dissociation curves following each run. The specificity of reactions had previously been confirmed by sequencing (Tu et al., 2019; Martínez et al., 2020). Gene expression was normalized using the NORMAgene approach (Heckmann et al., 2011) and expressed as fold-change of control group values.

**TABLE 1:** Primer sequences and annealing temperatures used in real-time RT-PCR assay for energy metabolism related gene targets.

Gene	NCBI Genbank ID	FW primer sequence	RV primer sequence	Tm (°C)
*apoa1a*	NM_131128.1	GAA​GGC​CTT​CGA​GTC​CAA​CA	TCT​GTG​CCG​AAT​GTG​GTC​CTC	55
*apoba*	XM_689735.9	AGC​TGA​AGA​ACG​CAC​TCT​CC	GAA​CTT​CAG​GGC​CGC​ATC​TA	57
*insa*	NM_131056.1	TAA​GCA​CTA​ACC​CAG​GCA​CA	GAT​TTA​GGA​GGA​AGG​AAA​CC	59
*insb*	NM_001039064.1	ACT​CTT​CAC​AGA​CTC​TGC​TC	ACA​GAT​GCT​GGG​ATG​GAG​AA	59
*pck*	NM_214751.1	GCA​CGG​AGT​GTT​TGT​AGG​G	GGT​CTC​GGT​TCA​GTT​CAC​G	56
*pomca*	NM_181438.3	GCC​CCT​GAA​CAG​ATA​GAG​CC	CTC​GTT​ATT​TGC​CAG​CTC​GC	54
*pomcb*	NM_001083051.1	TCC​ATC​GAG​CTC​CAA​AAC​CC	ACATTTTACGGTCTGCGT	54

### 2.5 Statistical Analysis

All data were analyzed using SPSS Version 27 and visualized using GraphPad Prism Version 8.0. Data were tested for normality and homoscedasticity using Shapiro-Wilk and Levene’s tests, respectively. In cases where data did not meet ANOVA criteria, standard transformations were used to improve normality and homoscedasticity of data. Univariate ANOVAs were used to assess significant effects of either treatment at a significance level of *p* < 0.05. In all cases cohorts were analyzed on individual plates and run as a fixed factor. Thus, the experimental design does not allow to dissociate (biological) cohort and (technical) plate effects. Significant differences in omnibus tests were resolved using Tukey’s post-hoc test at a significance level of *p* < 0.05. For repeated measurement data, the same between-subject factor (treatment) as above were used in analysis, in addition to within-subject factor (time). Significant between-subject and within-subject treatment effects and their interactions were assessed at a significance level of *p* < 0.05. In cases where the sphericity assumption was violated, as assessed using Mauchly’s W test, appropriate corrections (Greenhouse-Geisser or Huynh-Feldt) were used to assess significance of effects according to epsilon-based criteria as recommended (Girden, 1992).

## 3 Results

### 3.1 Nanoplastics Bioaccumulate in Zebrafish (eleuthero)embryos

**FIGURE 2:**
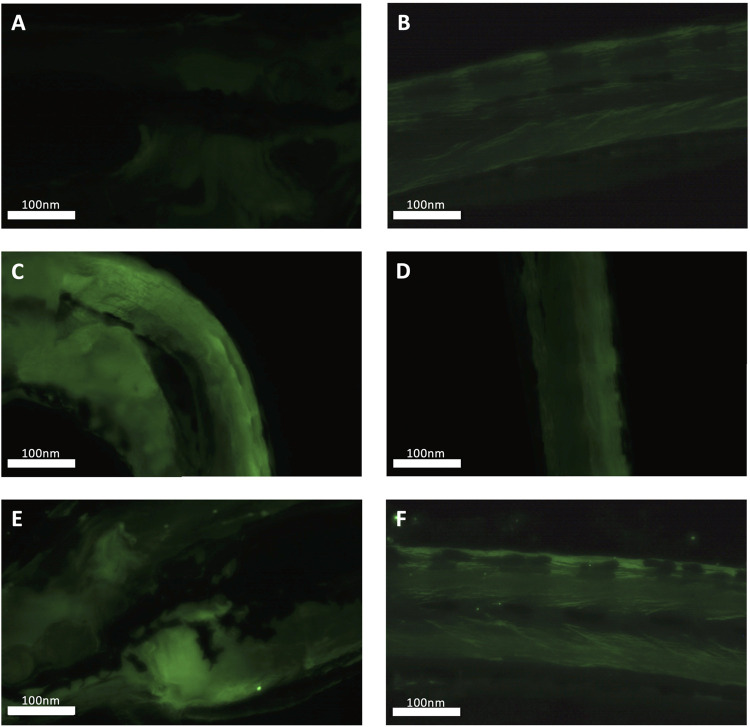
Fluorescence microscopy-based validation of nanoplastic uptake in control, 2.5 and 25 ppm fluorescent polystyrene nanoplastic particle exposed zebrafish. Representative head and tail images of control **(A,B)**, low PS **(C,D)** and high PS exposure group **(E,F)** 7 dpf zebrafish larvae are shown.

A qualitative increase in fluorescence signal was consistently observed in the PS nanoplastic exposed groups compared to the control group in the anterior part of eleutheroembryos containing the yolk sac and digestive tract, but not the caudal part containing skeletal muscle tissue (Figure 2).

### 3.2 Exposure to PS Nanoplastics, BDE-47 or Their Combination has Minimal Effects on Larval Growth

**FIGURE 3:**
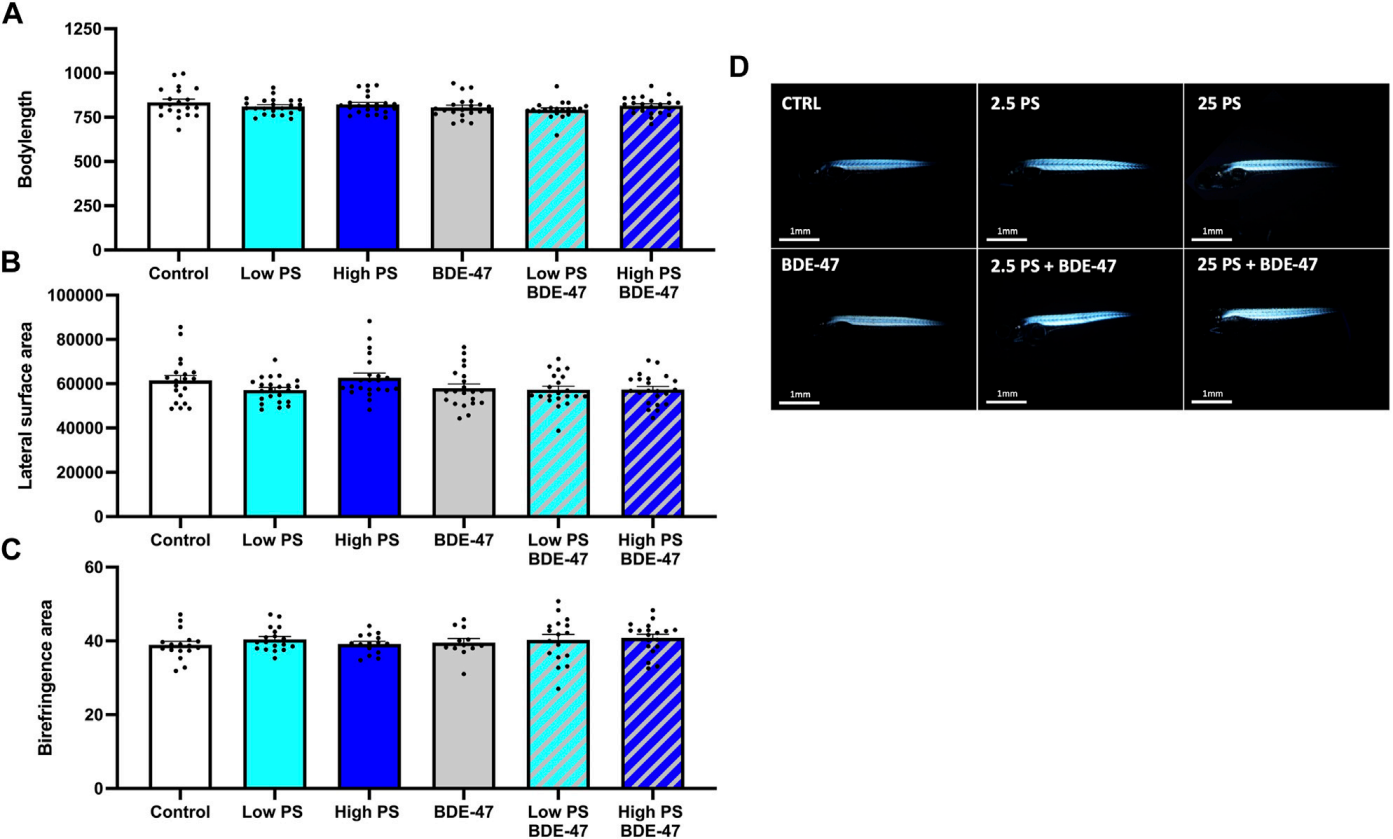
Morphometric indices of 7 dpf zebrafish larvae exposed to vehicle control, 2.5 ppm PS, 25 ppm PS, 10 ppt BDE-47 or their combinations: Body length **(A)**, lateral surface area **(B)** and birefringent lateral somite area **(C)**. Individual measurements and average data +S.E.M. from *n* = 3 replicate assays with combined *n* = 20–23 **(A,B)** and *n* = 12–19 **(C)** per treatment group are presented. Representative images of birefringence measurements of laterally positioned zebrafish are presented **(D)**. Data were analyzed using a one-way ANOVA with a significance cut-off of *p* < 0.05.

Treatment did not significantly affect body length (df = 5; F = 1.698; *p* = 0.141; Figure 3A). Treatment effect size (η2p) and observed power were 0.071 and 0.569, respectively. A significant cohort effect (df = 2; F = 34.937; *p* < 0.001) and significant interaction between cohort and treatment effect (df = 10; F = 2.037; *p* = 0.036) were observed. Treatment significantly affected lateral surface area (df = 5; F = 2.557; *p* = 0.031; Figure 3B). Treatment effect size (η2p) and observed power were 0.103 and 0.775, respectively. However, post-hoc analysis was unable to resolve differences between treatment groups. A significant cohort effect (df = 2; F = 26.008; *p* < 0.001) was observed. Muscle sarcomere-dependent birefringence was not affected by treatment (df = 5; F = 0.778; *p* = 0.561; Figure 3C). Treatment effect size (η2p) and observed power were 0.047 and 0.269, respectively. A significant cohort effect (df = 2; F = 17.711; *p* < 0.001) was observed. Representative images showing birefringence in each group are shown in Figure 3D.

### 3.2 PS Nanoplastics Alone and in Combination With BDE-47 Increase Larval Food Consumption

**FIGURE 4:**
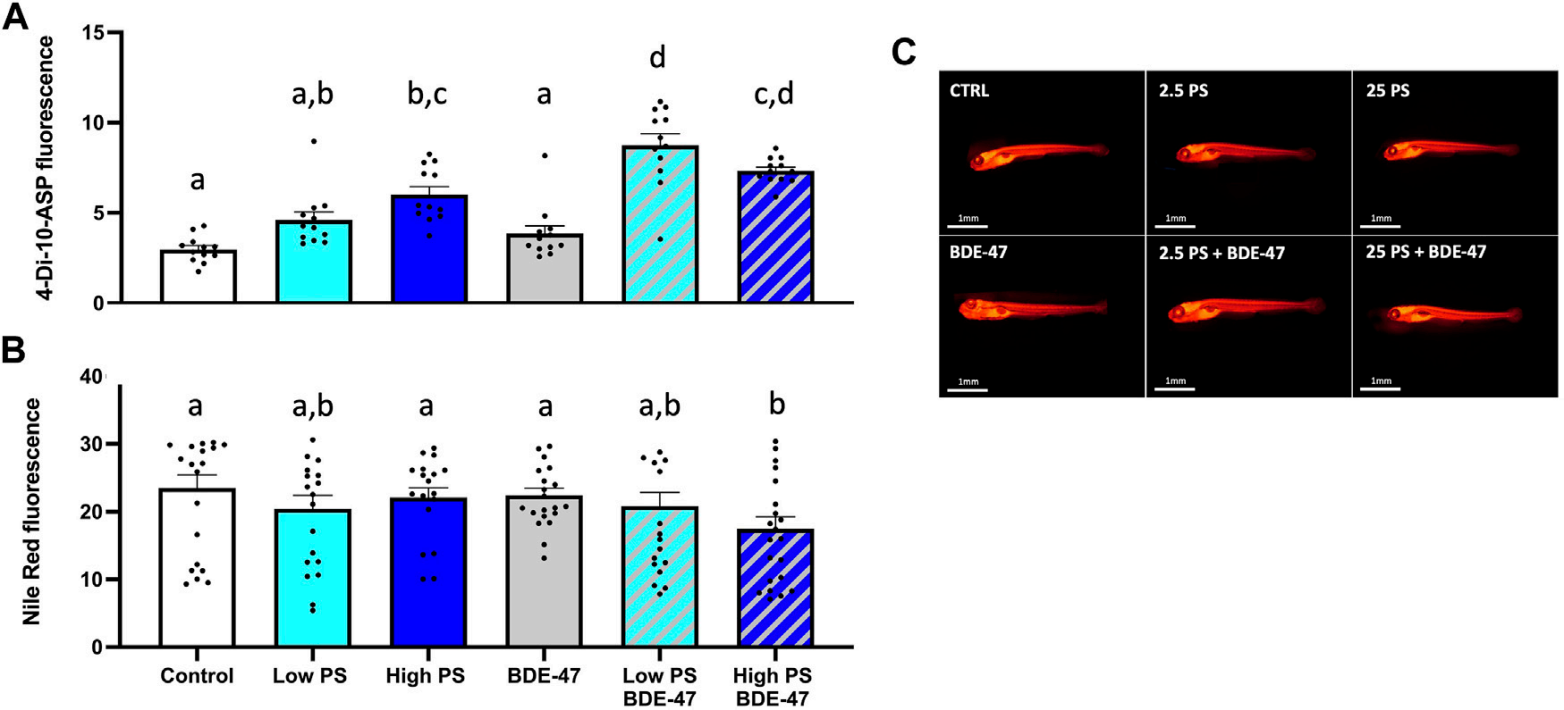
Feed-intake **(A)** and neutral lipid deposition **(B)** of 7 dpf zebrafish larvae exposed to vehicle control, 2.5 ppm PS, 25 ppm PS, 10 ppt BDE-47 or their combinations. Individual measurements and average data +S.E.M. from *n* = 3 replicate assays with a combined *n* = 12 **(A)** and *n* = 18–22 **(B)** per treatment group are presented. Representative images of Nile Red based neutral lipid staining are shown **(C)**. Data were analyzed using a one-way ANOVA with a significance cut-off of *p* < 0.05. Different letters indicate significant differences between groups as analyzed by Tukey’s post-hoc test.

Treatment significantly affected food consumption in 7 dpf larvae (df = 5; F = 35.256; *p* < 0.001; Figure 4A). The effect size (η2p) and observed power of the treatment were 0.766 and 1, respectively. A significant cohort effect (df = 2; F = 10.443; *p* < 0.001) was observed. Post-hoc analysis of the treatment effect revealed that PS exposure at both low and high concentrations alone and in co-exposure with BDE-47 significantly increased food consumption compared to control. Both low and high PS + BDE-47 co-exposures also significantly increased food consumption compared to BDE-47 exposure alone.

### 3.3 Co-Exposure to High PS Nanoplastic and BDE-47 Significantly Reduce Larval Neutral Lipid Stores

Treatment significantly affected neutral lipid storage in zebrafish larvae (df = 5; F = 11.274; *p* < 0.001; Figures 4B,C). The effect size (η2p) and observed power of the treatment were 0.34 and 1, respectively. Post-hoc analysis revealed a significant reduction of neutral lipid storage in zebrafish co-exposed to high PS + BDE-47 compared to control. A significant cohort effect (df = 2; F = 58.57; *p* < 0.001) was observed.

### 3.4 PS and BDE-47 Exposure and Their Combination Significantly Increase Oxygen Consumption Rates

**FIGURE 5:**
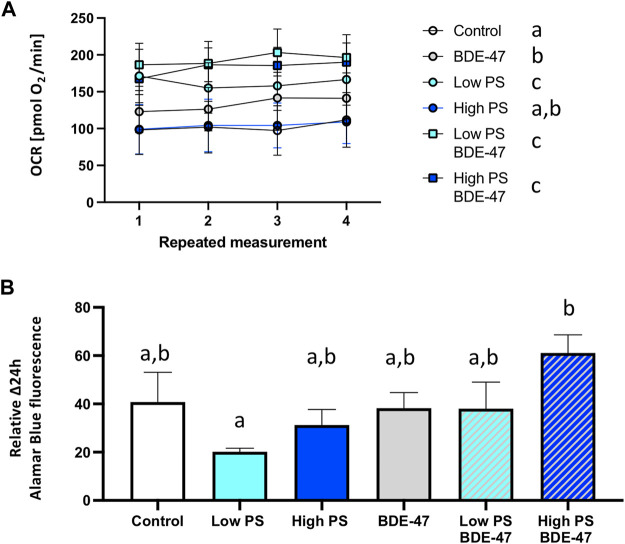
Oxygen consumption rate **(A)** and 24 h oxygen metabolism-dependent energy expenditure of 2 dpf zebrafish eleutheroembryos exposed to vehicle control, 2.5 ppm PS, 25 ppm PS, 10 ppt BDE-47 or their combinations **(B)**. Average oxygen consumption rates +S.E.M. from *n* = 3 replicate assays for each group with a combined *n* = 7–9 per treatment group are presented **(A)**. Average repeated measurements of 24 h oxygen metabolism-dependent energy expenditures +S.E.M. from *n* = 3 replicate assays for each group with an *n* = 33–47 per treatment group are presented **(B)**. Data were analyzed using a one-way repeated measurement ANOVA **(A)** and one-way ANOVA **(B)** measurement with a significance cut-off of *p* < 0.05. Different letters indicate significant differences between groups as analyzed by Tukey’s post-hoc test.

Treatment significantly affected oxygen consumption in 2 dpf zebrafish (df = 5; F = 142.6; *p*=<0.001; Figure 5A). The effect size (η2p) and observed power of the treatment were 0.965 and 1.0, respectively. Post-hoc analysis revealed that all treatments except 25 ppm PS significantly increased oxygen consumption rate (OCR) compared to controls. Irrespective of the PS concentration used, co-exposure groups exhibited increased OCR compared to BDE-47 exposed fish alone. In the high PS + BDE-47 co-exposure group, this increase was also significant compared to the high PS exposure alone. No significant cohort effect (df = 3; F = 2.765 *p* < 0.059) was observed. Treatment significantly affected 24 h oxidative metabolism related energy expenditure in 2 dpf zebrafish as quantified using the Alamar Blue assay (df = 5; F = 2.918; *p* = 0.014; Figure 5B). The effect size (η2p) and observed power of the treatment were 0.06 and 0.847, respectively. Post-hoc analysis revealed a significantly increased oxidative metabolism related energy expenditure in eleutheroembryos exposed to high PS + BDE-47 compared to elutheroembryos exposed to low PS alone. No significant cohort effect (df = 2; F = 1.331; *p* < 0.266) was observed.

### 3.5 Locomotor Assay

**FIGURE 6:**
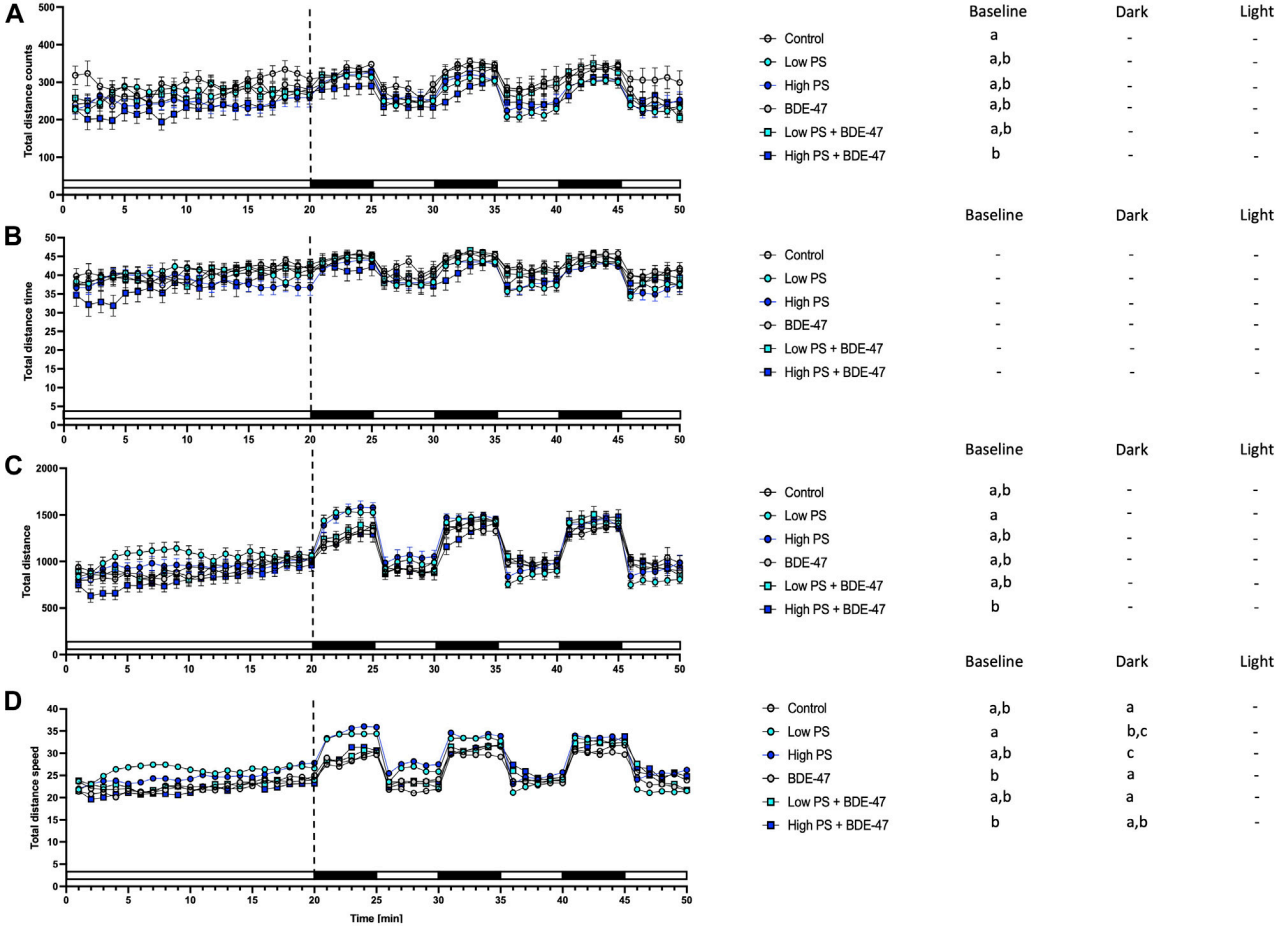
Total movement parameters from 7 dpf zebrafish larvae locomotion assays (*n* = 3) with a total *n* = 48 per treatment group consisting of exposures to vehicle control, 2.5 ppm PS, 25 ppm PS, 10 ppt BDE-47 or their combinations. All measurements over the 50 min assays were binned in 1 min units, with 20 min baseline followed by 3 cycles of 5 min dark light alterations. Average total counts of movement events +/− S.E.M. **(A)**, average duration of total movement events +/− S.E.M in s **(B)**, Distance of total movement events +/− S.E.M in mm **(C)**, Average speed +/− S.E.M of total movements in mm/s **(D)**. Data were analyzed using separate one-way repeated measurement ANOVAs for baseline, dark, and light conditions. Significant treatment effects (*p* < 0.05) are shown in Table 2, and specific post-hoc analysis results are depicted next to the graphs for all parameters. Different letters indicate significant differences between groups as analyzed by Tukey’s post-hoc test (*p* < 0.05).

Several indices of locomotion were affected by treatment under baseline conditions in zebrafish larvae (Table 2). Cohort effects are reported in Table 3. Counts of total movement observations were significantly reduced in zebrafish larvae co-exposed to high PS + BDE-47 compared to control (*p* < 0.05; Figure 6A). While total movement duration did not exhibit significant differences between treatment groups (Figure 6B), total distance covered (Figure 6C) and total movement speed (Figure 6D) were significantly affected by treatment. However, post-hoc analysis revealed that no treatment group exhibited significant differences from the control group. When analyzing parameters under specific lighting conditions, zebrafish larvae under dark conditions exhibited specific responses to treatment both for total movement counts (Figure 6A) and total movement speed (Figure 6D). However, only total movement speed in low and high PS exposure groups revealed significant increases compared to control group (*p* < 0.05; Figure 6D).

**TABLE 2:** Repeated measurement ANOVA analyses of treatment effects on locomotion assay endpoints. Effect sizes and observed power are reported. Bold font indicates significance at a *p* < 0.05 threshold.

Locomotion endpoint	Baseline	Dark	Light
Total movement counts	**df = 5** **F = 2.641 *p* = 0.024**	**df = 5** **F = 2.509 *p* = 0.031**	df = 5 F = 2.246 *p* = 0.050
	*η2p = 0.47 Power = 0.80*	*η2p = 0.44 Power = 0.78*	*η2p = 0.40 Power = 0.73*
Total movement duration	df = 5 F = 0.896 *p* = 0.485	df = 5 F = 0.765 *p* = 0.575	df = 5 F = 1.828 *p* = 0.108
	*η2p = 0.16 Power = 0.32*	*η2p = 0.14 Power = 0.27*	*η2p = 0.33 Power = 0.62*
Total movement distance	**df = 5** **F = 2.509 *p* = 0.031**	df = 5 F = 1.747 *p* = 0.124	df = 5 F = 0.461 *p* = 0.805
	*η2p = 0.44 Power = 0.78*	*η2p = 0.31 Power = 0.60*	*η2p = 0.08 Power = 0.17*
Total movement speed	**df = 5** **F = 3.216 *p* = 0.008**	**df = 5 F = 9.623 *p* = 0.001**	df = 5 F = 1.090 *p* = 0.366
	*η2p = 0.57 Power = 0.89*	*η2p = 0.15 Power = 1.00*	*η2p = 0.20 Power = 0.39*
Short movement counts	**df = 5** **F = 2.898 *p* = 0.014**	**df = 5** **F = 3.479 *p* = 0.005**	**df = 5** **F = 2.465 *p* = 0.044**
	*η2p = 0.05 Power = 0.84*	*η2p = 0.06 Power = 0.91*	*η2p = 0.04 Power = 0.77*
Short movement duration	**df = 5** **F = 3.161 *p* = 0.009**	**df = 5** **F = 13.41 *p* = 0.001**	**df = 5** **F = 6.236 *p* = 0.001**
	*η2p = 0.06 Power = 0.88*	*η2p = 0.20 Power = 1.00*	*η2p = 0.10 Power = 1.00*
Short movement distance	**df = 5** **F = 4.411 *p* = 0.001**	**df = 5 F = 14.39 *p* = 0.001**	**df = 5** **F = 7.163 *p* = 0.001**
	*η2p = 0.08 Power = 0.97*	*η2p = 0.21 Power = 1.00*	*η2p = 0.12 Power = 1.00*
Short movement speed	**df = 5** **F = 6.876 *p* = 0.001**	**df = 5** **F = 5.081 *p* = 0.001**	**df = 5** **F = 4.081 *p* = 0.001**
	*η2p = 0.12 Power = 1.00*	*η2p = 0.09 Power = 0.98*	*η2p = 0.07 Power = 0.95*
Long movement counts	**df = 5** **F = 2.259 *p* = 0.049**	df = 5 F = 1.727 *p* = 0.129	df = 5 F = 2.090 *p* = 0.067
	*η2p = 0.04 Power = 0.73*	*η2p = 0.03 Power = 0.59*	*η2p = 0.04 Power = 0.69*
Long movement duration	**df = 5** **F = 2.270 *p* = 0.048**	**df = 5** **F = 4.721 *p* = 0.001**	df = 5 F = 0.996 *p* = 0.421
	*η2p = 0.04 Power = 0.73*	*η2p = 0.08 Power = 0.98*	*η2p = 0.02 Power = 0.35*
Long movement distance	**df = 5** **F = 4.929 *p* = 0.001**	**df = 5** **F = 6.123 *p* = 0.001**	df = 5 F = 1.495 *p* = 0.191
	*η2p = 0.08 Power = 0.98*	*η2p = 0.10 Power = 1.00*	*η2p = 0.03 Power = 0.52*
Long movement speed	**df = 5** **F = 3.654 *p* = 0.003**	df = 5 F = 1.398 *p* = 0.255	df = 5 F = 0.709 *p* = 0.617
	*η2p = 0.07 Power = 0.93*	*η2p = 0.03 Power = 0.49*	*η2p = 0.01 Power = 0.26*

**TABLE 3:** Cohort effects in repeated measurement ANOVA analyses on locomotion assay endpoints. Bold font indicates significance at a *p* < 0.05 threshold.

Locomotion endpoint	Baseline	Dark	Light
Total movement counts	**df = 2** **F = 5.013 *p* = 0.007**	df = 2 F = 0.870 *p* = 0.420	df = 2 F = 0.637 *p* = 0.529
Total movement duration	**df = 2** **F = 4.462 *p* = 0.012**	df = 2 F = 0.120 *p* = 0.887	df = 2 F = 0.61 *p* = 0.941
Total movement distance	**df = 2** **F = 10.10 *p* = 0.001**	**df = 2** **F = 6.358 *p* = 0.002**	**df = 2** **F = 5.871 *p* = 0.003**
Total movement speed	**df = 2** **F = 6.287 *p* = 0.002**	**df = 2** **F = 22.63 *p* = 0.001**	df = 2 F = 1.169 *p* = 0.312
Short movement counts	**df = 2** **F = 4.578 *p* = 0.011**	df = 2 F = 1.013 *p* = 0.365	df = 2 F = 0.999 *p* = 0.370
Short movement duration	**df = 2** **F = 1.763 *p* = 0.174**	**df = 2** **F = 10.63 *p* = 0.001**	**df = 2** **F = 8.238 *p* = 0.001**
Short movement distance	df = 2 F = 2.620 *p* = 0.075	**df = 2** **F = 12.105 *p* = 0.001**	**df = 2** **F = 9.279 *p* = 0.001**
Short movement speed	**df = 2** **F = 4.058 *p* = 0.018**	df = 2 F = 2.596 *p* = 0.077	**df = 2** **F = 3.525 *p* = 0.031**
Long movement counts	**df = 2** **F = 5.141 *p* = 0.006**	df = 2 F = 1.100 *p* = 0.335	df = 2 F = 0.618 *p* = 0.540
Long movement duration	**df = 2** **F = 7.918 *p* = 0.001**	**df = 2** **F = 7.074 *p* = 0.001**	**df = 2** **F = 8.923 *p* = 0.001**
Long movement distance	**df = 2** **F = 12.283 *p* = 0.001**	**df = 2** **F = 14.594 *p* = 0.001**	**df = 2** **F = 11.56 *p* = 0.001**
Long movement speed	**df = 2** **F = 13.72 *p* = 0.001**	**df = 2** **F = 13.79 *p* = 0.001**	df = 2 F = 1.959 *p* = 0.143

**FIGURE 7:**
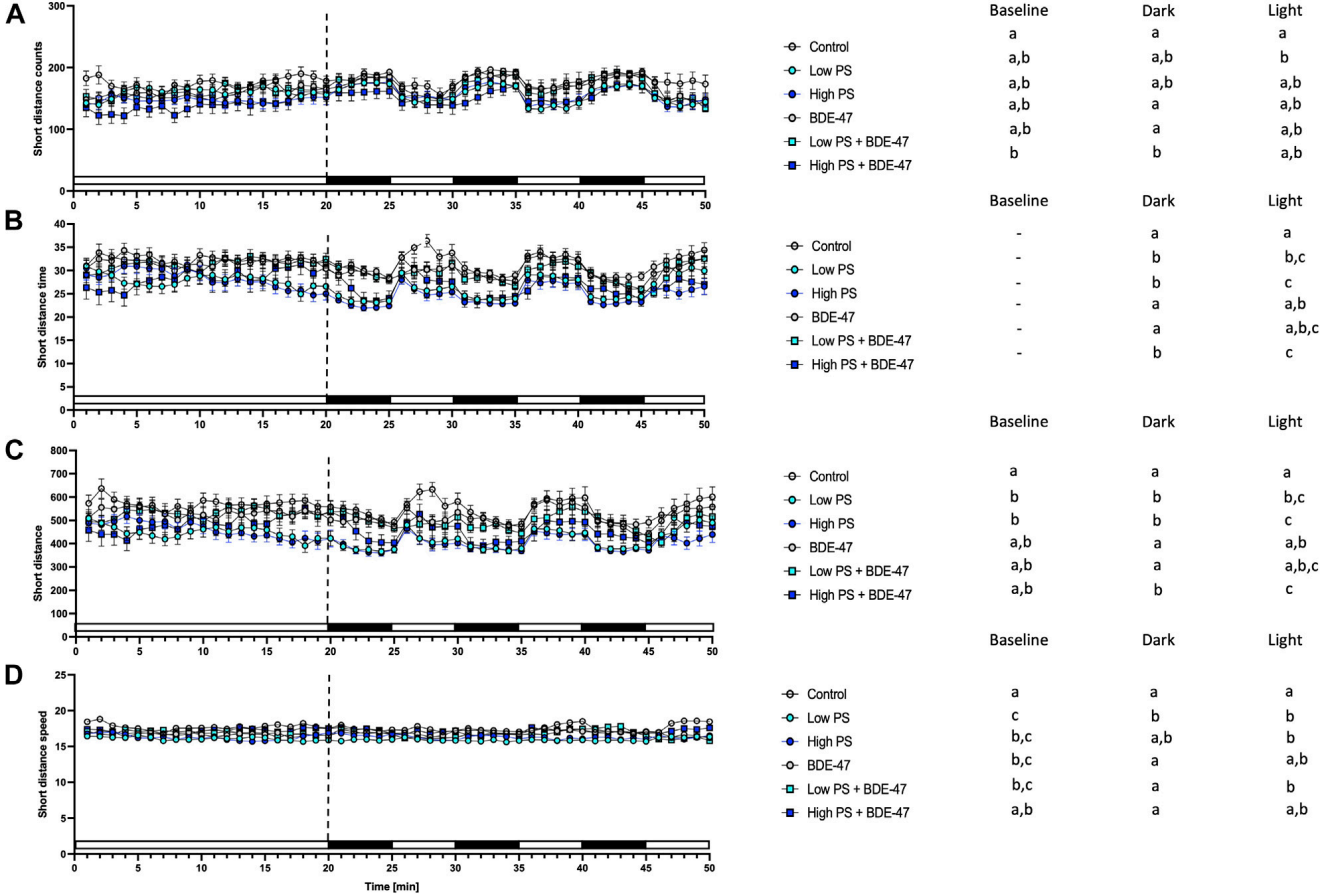
Short movement parameters from 7 dpf zebrafish larvae locomotion assays (*n* = 3) with a total *n* = 48 for each treatment groups. All measurements over the 50 min assays were binned in 1 min units, with 20 min baseline followed by 3 cycles of 5 min dark light alterations. Average short counts of movement events +/− S.E.M. **(A)**, average duration of total movement events +/− S.E.M in s **(B)**, Distance of short movement events +/− S.E.M in mm **(C)**, Average speed +/− S.E.M of short movements in mm/s **(D)**. Data were analyzed using separate one-way repeated measurement ANOVAs for baseline, dark, and light conditions. Significant treatment effects (*p* < 0.05) are shown in Table 1, and specific post-hoc analysis results are depicted next to the graphs for all parameters. Different letters indicate significant differences between groups as analyzed by Tukey’s post-hoc test (*p* < 0.05).

Indices of short movement were affected by treatment in zebrafish larvae under baseline conditions (Table 2). Counts of short movement were significantly decreased in zebrafish co-exposed to high PS and BDE-47 compared to control (*p* < 0.05; Figure 7A). While significant effects of treatment on short movement duration could not be resolved by post-hoc analysis (Figure 7B), short movement distance (Figure 7C) and short movement speed (Figure 7D) exhibited significant reductions in low and high PS compared to control (*p* < 0.05; Figure 7C) and in all treatment groups except for high PS + BDE-47 compared to control (*p* < 0.05; Figure 7D), respectively. All measured short distance parameters were also significantly affected by treatment when analyzing dark and light conditions (Table 2). In dark conditions, short movement counts were significantly reduced in larvae exposed to high PS + BDE-47 compared to control (*p* < 0.05; Figure 7A). Short movement distance covered in low PS, high PS and high PS + BDE-47 was significantly less compared to control (*p* < 0.05; Figure 7C), while short movement speed was significantly reduced by low PS exposure compared to control (*p* < 0.05; Figure 7D) under dark conditions. In light conditions, short movement counts were significantly reduced in the low PS exposure group compared to control (*p* < 0.05; Figure 7A), while short movement duration and distance were significantly lower in low PS, high PS and high PS + BDE-47 exposed groups compared to control (*p* < 0.05; Figures 7B,C). Under light conditions short movement speed was slower in low PS, high PS and low PS + BDE-47 exposed groups compared to control (*p* < 0.05; Figure 7D).

**FIGURE 8:**
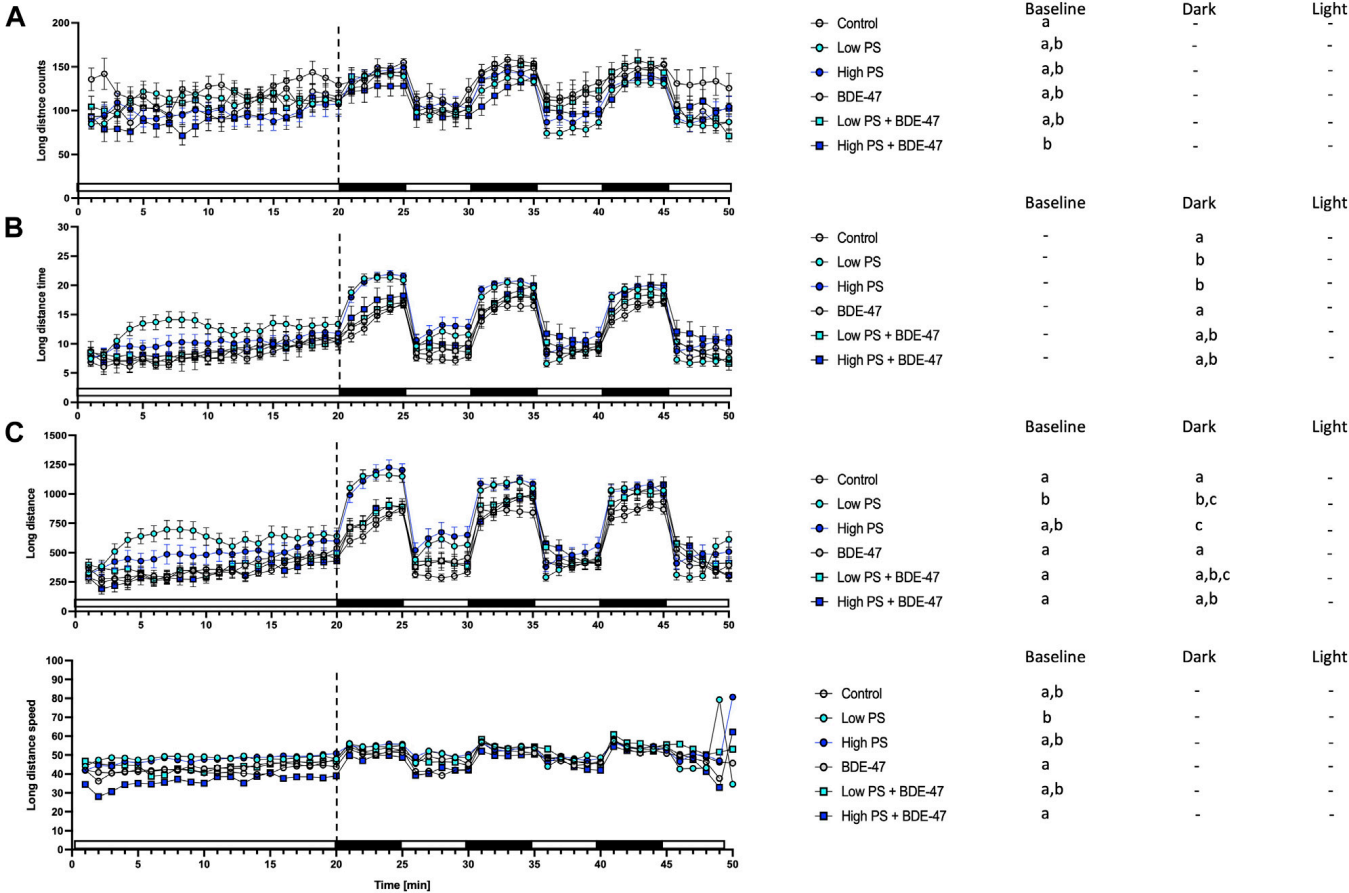
Long movement parameters from 7 dpf zebrafish larvae locomotion assays (*n* = 3) with a total *n* = 48 for each treatment group consisting of exposures to vehicle control, 2.5 ppm PS, 25 ppm PS, 10 ppt BDE-47 or their combinations. All measurements over the 50 min assays were binned in 1 min units, with 20 min baseline followed by 3 cycles of 5 min dark light alterations. Average long counts of movement events +/− S.E.M. **(A)**, average duration of total movement events +/− S.E.M in s **(B)**, Distance of long movement events +/− S.E.M in mm **(C)**, Average speed +/− S.E.M of long movements in mm/s **(D)**. Data were analyzed using separate one-way repeated measurement ANOVAs for baseline, dark, and light conditions. Different letters indicate significant differences between groups as analyzed by Tukey’s post-hoc test (*p* < 0.05).

Indices of short movement were affected by treatment in zebrafish larvae under baseline conditions (Table 2). Under baseline conditions, counts of long distance movement were significantly decreased in high PS + BDE-47 exposed larvae compared to control (*p* < 0.05; Figure 8A). Conversely low PS exposed larvae covered larger distances with long movement at greater speed compared to control larvae (*p* < 0.05; Figures 8C,D). Under dark conditions, low and high PS exposure increased duration and distance of long movements compared to controls (*p* < 0.05; Figures 8B,C).

### 3.6 Gene Expression

**FIGURE 9:**
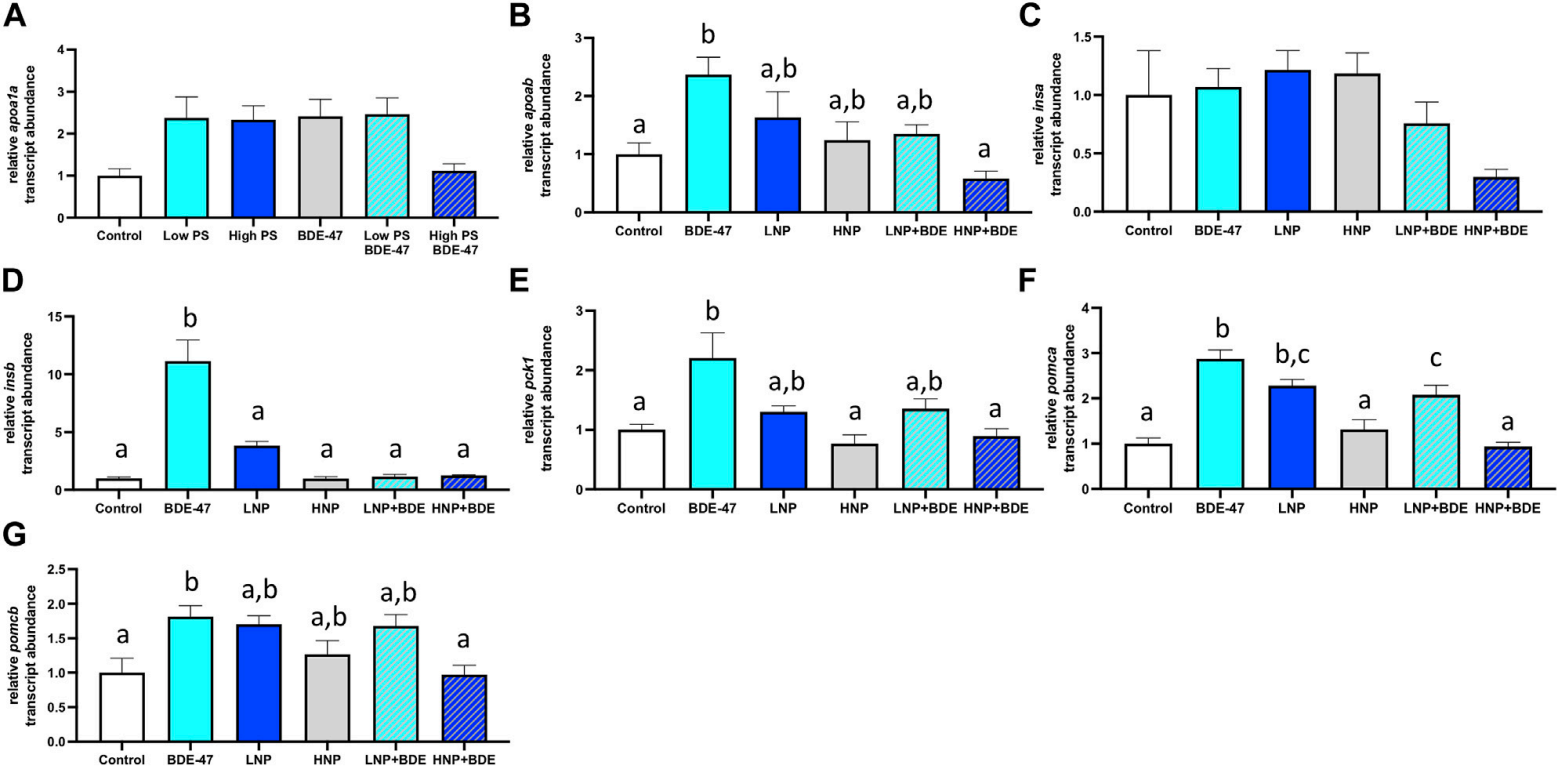
Relative abundance of mRNA transcripts with characterized roles in energy metabolism in whole 7 dpf larvae. Treatment groups consist of exposures to vehicle control, 2.5 ppm PS, 25 ppm PS, 10 ppt BDE-47 or their combinations. Apolipoprotein transcripts *apoa1a* **(A)** and *apoab* **(B)**, insulin paralogue transcripts *insa* **(C)** and *insb* **(D)**, the gluconeogenic marker *pck1* **(E)** and *pomc* paralogues *pomca* **(F)** and *pomcb* **(G)**. Average mRNA transcript abundance +S.E.M. of *n* = 4 pooled replicates per treatment group containing *n* = 10 larvae per replicate are shown. Relative transcript abundance was normalized to control group to reflect fold-change. All data was analyzed using one-way ANOVAs with a significance cut-off of *p* < 0.05. Where omnibus tests revealed a significant treatment effect, Tukey’s post-hoc test (*p* < 0.05) was used to discern significant differences between treatment groups, which are indicated by different letters.

Relative transcript abundance of *apoa1a* was significantly affected by treatment (df = 5; F = 3.936; *p* = 0.0138; Figure 9A). The effect size (η2p) and observed power of the treatment were 0.52 and 0.86, respectively. However, Tukey’s post-hoc analysis was unable to discern differences in *apoa1a* transcript abundance between specific treatment groups. Relative transcript abundance of *apoba* was significantly affected by treatment (df = 5; F = 4.786; *p* = 0.0059; Figure 9B). The effect size (η2p) and observed power of the treatment were 0.57 and 0.92, respectively. Tukey’s post-hoc analysis revealed a significant increase of *apoa1a* transcripts in BDE-47 exposed zebrafish compared to control and BDE-47 co-exposed with high PS nanoplastics (*p* < 0.05). While relative transcript abundance of *insa* was not significantly affected by treatment (df = 5; F = 2.709; *p* = 0.0539; Figure 9C), *insb* transcript abundance was significantly affected by treatment (df = 5; F = 25.52; *p* < 0.0001; Figure 9D). The effect size (η2p) and observed power of the treatment were 0.43 and 0.68 for *insa* and 0.88 and 1.00 for *insb*. Post-hoc analysis revealed that BDE-47 significantly induced *insb* transcript abundance compared to all other treatment groups. Relative transcript abundance of *pck1* was similarly affected by treatment (df = 5; F = 6.293; *p* = 0.0015; Figure 9E), which post-hoc analysis resolved as significant increase in *pck1* transcript abundance in BDE-47 exposed zebrafish compared to control, high PS nanoplastics and co-exposure to high PS nanoplastics and BDE-47 (*p* < 0.05). The effect size (η2p) and observed power of the treatment were 0.64 and 0.98. Relative transcript abundance of *pomca* was significantly altered by treatment (df = 5; F = 21.24; *p* < 0.0001; Figure 9F), with significantly increased expression in BDE-47, low PS nanoplastic and low PS nanoplastic + BDE-47 exposed zebrafish compared to controls (*p* < 0.05). The effect size (η2p) and observed power of the treatment were 0.86 and 1.00. Relative transcript abundance of *pomcb* responded to treatment (df = 5; F = 4.932; *p* = 0.0051; Figure 9G), with significant increase in *pomcb* expression in BDE-47 exposed zebrafish compared to control and BDE-47 + high PS nanoplastic co-exposure. The effect size (η2p) and observed power of the treatment were 0.96 and 1.00.

## 4 Discussion

### 4.1 PS Nanoplastics Accumulate in Exposed Zebrafish

A qualitative increase in fluorescence-labelled PS nanoplastics was observed especially in the anterior part of eleutheroembryos at both 2.5 and 25 ppm exposure concentrations, confirming uptake. In contrast to several studies, our study used a dialysis protocol before PS exposure, following reports of fluorescent dye leaching which may result in artifacts during imaging (Schür et al., 2019; Xu et al., 2019). Discernable increases in signal intensity were observed in ventral regions containing the digestive tract at both 2.5 ppm and 25 ppm PS nanoplastic exposure concentrations. At the 25 ppm concentration, fluorescence-labelled PS nanoplastics, albeit to a lesser extent, were also detected in the tail region. To assess fluorescence labelled NP uptake at the tissue level, we exposed adult zebrafish to 25 ppm PS for 4 days. Subsequent histological analysis revealed increased fluorescence in the intestinal tract and liver, both of which also exhibited potential signs of inflammation such as enlarged goblet cells and hyperemia, respectively (Supplementary Figure S1).

These findings are in line with previous reports confirming uptake of PS nanoplastics principally in the intestine (Lu et al., 2016; Skjolding et al., 2017; Pitt et al., 2018; Brun et al., 2019; Trevisan et al., 2019; Trevisan et al., 2020). Oral ingestion following hatching has been characterized as the principal route of exposure in developing zebrafish, with limited biodistribution following ingestion for PS nanoparticles larger than 50 nm in size (van Pomeren et al., 2017). Together, these findings confirm PS nanoplastic uptake of 100 nm particles occurs especially in the dorsovisceral region of 7 dpf zebrafish in our study. While BDE-47 uptake or sorption to PS nanoplastics was not quantified in the current study, BDE-47 has, due to its high *K*_OW_ of >6.5, been shown to bioaccumulate in zebrafish embryos in previous studies with reported BCF exceeding 2000 (Zheng et al., 2012; Usenko et al., 2013; Liu et al., 2015). BDE-47 has also been shown to sorb to nanoplastics, especially PS (Xu et al., 2019; Horton et al., 2020; Wu et al., 2020). While our experimental design should ensure that both compounds are taken up in early developing zebrafish, the lack of analytical assessment of internal BDE-47 concentration measurement across treatment groups does not allow us to determine whether the 100 nm PS nanoplastic serve as a vector to increase internal BDE-47 concentration via ingestion, as reported for 20 nm PS nanoplastics and POPs (Zhang and Goss, 2020) or reduce BDE-47 uptake by sequestering free BDE-47 in the medium, as reported for 5 μm PS microplastics (Yang et al., 2020).

### 4.2 Individual PS Nanoplastic and BDE-47 Exposure Induces Organismal-Level Metabolic Changes in Early Zebrafish Development

**FIGURE 10:**
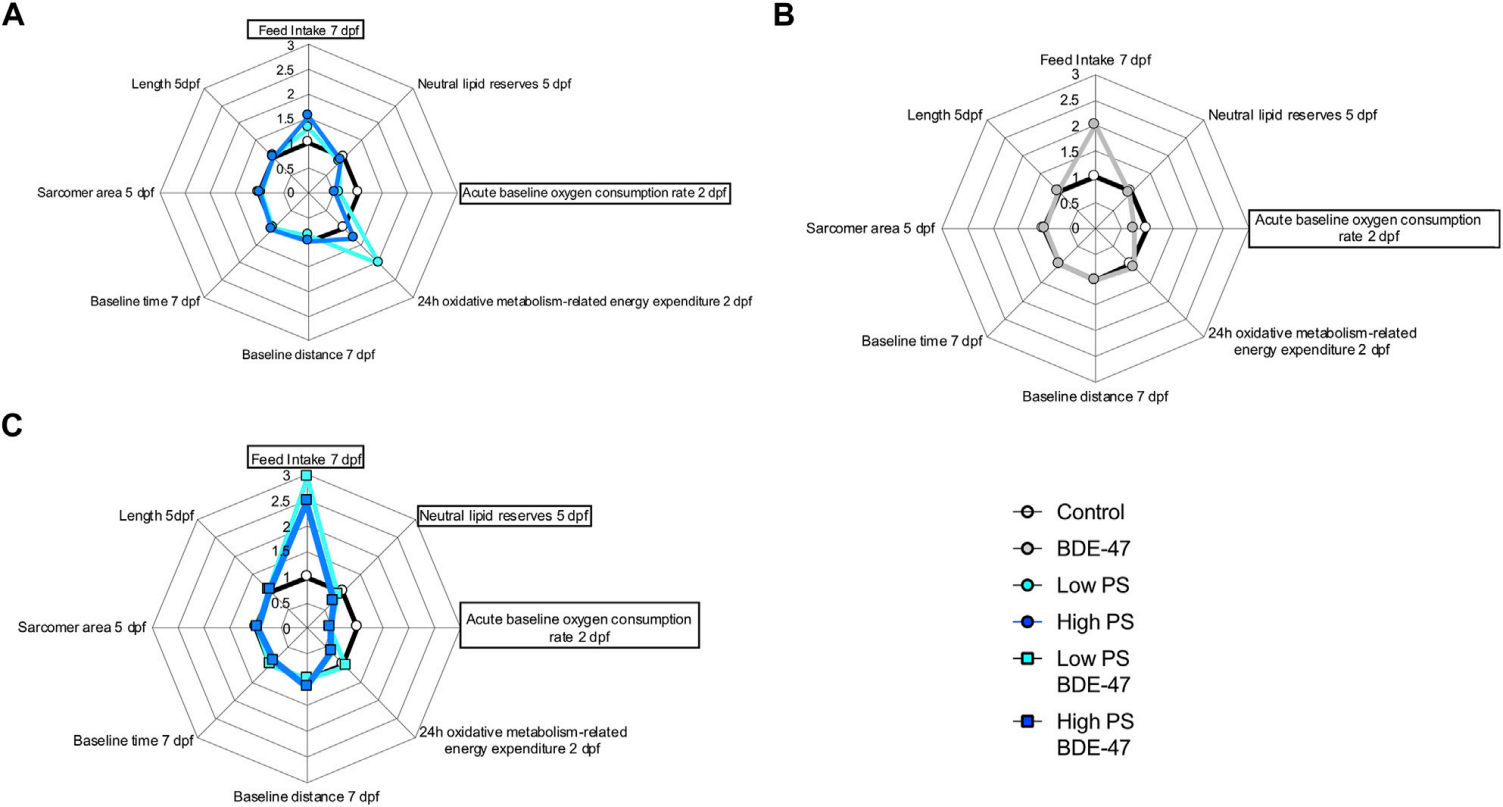
Radar chart diagrams summarizing organismal level effects of different exposure treatments on the metabolic phenotype in developing zebrafish: Control, 2.5 ppm PS and 25 ppm PS exposures **(A)** Control and 10 ppt BDE-47 exposure, **(B)** Control and combined exposures of 2.5 ppm + 10 ppt BDE-47 and 25 ppm + 10 ppt BDE-47, **(C)** in comparison to vehicle control. Note that all average treatment group values have been normalized to control values (=1) and in some cases inverted so that datapoints >1 indicate quantified parameters with a positive contribution to energy balance, while datapoints <1 indicate parameters negatively contributing to organismal energy balance. Endpoints with significant effects of treatment are highlighted by surrounding rectangular shapes.

Developing zebrafish exposed to PS nanoplastics as well as BDE-47 exhibit similar metabolic effects at the organismal level. Individual exposure to both compounds increased oxygen consumption rate at 2 dpf, a developmental window already characterized by high oxygen demand (Figure 10). An increase in oxygen consumption rate indicates an increased demand for oxidative metabolism to cover energetic needs. Higher energetic needs, in turn, may be linked to developmental timing (Figure 10), and increased cost of detoxification responses (Handy et al., 1999; Scott and Sloman, 2004), but may also reflect direct contaminant disruption of mitochondrial efficiency and ROS buffering (Souders et al., 2018; Yang et al., 2021). At the internal respiration level, 10 ppm PS nanoplastic exposure has recently been shown to reduce coupling efficiency in developing zebrafish mitochondria at 2 and 4 dpf, without, however affecting baseline oxygen consumption rate (Trevisan et al., 2019). In the same study, a concurrent increase in NADH equivalents was reported, suggested to constitute either a consequential build-up of reducing equivalents in response to decreased coupling efficiency and/or a compensatory response driven by increased production of NADH equivalents. Metabolite level analysis provides further support PS induced mitochondrial uncoupling, as adult zebrafish exposure to 1.5 ppm PS nanoplastics resulted in increased ROS and decreased ATP concentrations in muscle tissues. Conversely, the increase in oxygen consumption rate was not accompanied by significant alterations of reduction equivalents in our study, although a strong tendency for a decrease was observed especially in the low PS exposed group. It is important to note that the Alamar Blue assay used to assess reducing equivalents fluorometrically quantifies both NADH and NADPH and is therefore sensitive to detoxification and oxidative stress responses fuelled by NADPH (Tu et al., 2019; Wu et al., 2019; Martínez et al., 2020). Indeed, robust induction of oxidative stress and detoxification responses have been reported for PS in developing zebrafish (Hu and Palić, 2020). Future studies should thus extend the Seahorse assay to probe a contribution of PS induced disruption of mitochondrial coupling efficiency and probe markers of oxidative stress such as glutathione lipid peroxidation or antioxidant gene expression and activity to differentiate between these two distinct possibilities. Irrespective of the key mechanism driving increased oxygen demand, however, we observe a concurrent increase in feeding rate, an organismal level response likely to meet increased demand for oxidizable fuel to provide ATP via mitochondrial respiration. Since PS nanoplastics are, following hatching, ingested and quantified in the intestinal tract and key tissues involved in the regulation of energy metabolism such as the liver and pancreas (van Pomeren et al., 2017; Pitt et al., 2018), it is possible that reduced nutrient absorption and metabolism, as well as disrupted endogenous nutrient sensing, may contribute to the increase in feeding rate (Jovanović, 2017). Nevertheless, these organismal level metabolic changes did not translate into altered growth as quantified by body length lateral surface area and sarcomere development, suggesting that at least in early life stages, zebrafish (eleuthero)embryos and larvae can mount sufficient homeostatic responses in response to 2.5 and 25 ppm PS exposure to maintain somatic growth. At the behavioural level, overall locomotion was generally not affected by PS exposure, indicating that metabolic challenges did also not necessitate compensatory hypolocomotory behaviour to conserve energy. However some exceptions to these general findings exist, such a significant increase in locomotor speed under dark conditions. These findings are in contrast to previous findings demonstrating a hypo- (Chen et al., 2017; Qiang and Cheng, 2019) or hyperlocomotory (Brun et al., 2019) phenotype in zebrafish larvae following PS nanoplastic exposure, albeit at higher, mg/L exposure concentrations and a smaller diameter. Interestingly, separation of short and long movement bouts revealed that while total locomotory behaviour remained largely unaffected, indices of short movement (distance, time, speed) decreased, while the same indices increased for long movements under both baseline and different lighting conditions. Thus, while overall locomotion remains largely unaffected, the nature of locomotion exhibits specific responses to PS exposure, prioritizing long over short movements. Such changes may reflect foraging behaviour in line with increased feeding rates but may also be the consequence of peripheral feedback modulation or central control of locomotory behaviour, especially given that PS nanoplastics have been shown to affect lateral neuromasts in zebrafish (Brun et al., 2018) and have been shown to accumulate in the brain and muscle tissue where they have been shown to affect acetylcholine metabolism, concentration and neurons (Ding et al., 2018; Sarasamma et al., 2020; Yang et al., 2021). An integrated overview of the organismal level phenotypic consequences of PS exposure is presented in Figure 10A.

Similar to the PS exposure groups, BDE-47 exposure significantly increased oxygen consumption rate compared to control zebrafish (eleuthero)embryos. As previously discussed for PS, this may reflect higher energetic cost linked to detoxification and ROS buffering responses, as robust BDE-47 induced oxidative stress has been reported in a variety of species including (developing) zebrafish (Fernie et al., 2005; Shao et al., 2008; Tagliaferri et al., 2010; Costa et al., 2015; Usenko et al., 2015; Meng et al., 2020; Messina et al., 2020). Again, similar to described PS mode of actions, BDE-47 and its hydroxylated metabolite 6-OH BDE-47, have recently been described to decrease mitochondrial OXPHOS gene expression and ATP production and increase oxygen consumption indicative of OXPHOS disruption uncoupling in zebrafish (Legradi et al., 2017; Zhuang et al., 2020), suggesting a mitochondrial contribution to the observed increase in oxygen consumption rate. Like PS, BDE-47 exposure also increased the feeding rate. Increases in feeding in response to BDE-47 have recently also been reported in a marine rotifer, *Brachionus plicatilis*, where it has been linked to digestive suppression following BDE-47 induced mitochondrial disruption affecting cilia development on the one hand, and acetylcholine dependent control of feeding behaviour on the other (Yang et al., 2021). BDE-47 may promote zebrafish feed-intake as compensatory consequences of decreased nutrient absorption or mitochondrial function, or directly via disruption of central feeding circuits. The concomitant increase in oxygen consumption rate in response to BDE-47 exposure suggests that increased feeding rates may represent a homeostatic response to provide oxidizable fuel for energetically costly detoxification or mitochondria-derived ROS buffering processes widely reported for BDE-47 (Fernie et al., 2005; Shao et al., 2008; Tagliaferri et al., 2010; Costa et al., 2015; Usenko et al., 2015; Meng et al., 2020; Messina et al., 2020). However, possible directly inhibitory effects of BDE-47 on nutrient absorption and/or central suppression on feed-intake may represent alternative modes of action. Interestingly, cilia disruption has also been reported in zebrafish in response to other halogenated POPs such as PFOS (Huang et al., 2021), and histological examination of intestinal cell integrity including cilia development are warranted to probe potential effects on nutrient absorption. With regard to potential direct central effects of BDE-47 on feeding circuits, it is interesting to note that BDE-47 exposure decreased 5-HT-ir neurons in the zebrafish brain, a known anorexigenic factor in fish (Mennigen et al., 2009). In male mice, a 4 weeks exposure to 1 or 10 mg/kg/d BDE-47 resulted in ER-dependent alterations of several peptides involved in the regulation of feed intake and energy expenditure in the arcuate nucleus (Krumm et al., 2018). Overt effects on morphometric indices of growth were not observed, in line with reported findings of developmental delays or morphological effects of BDE-47 in zebrafish occur only at concentrations an order of magnitude higher than concentrations used in our experiment (Lema et al., 2007). Locomotory behaviour was unaffected by BDE-47 exposure, with the exception of reducing small movement speed under baseline conditions compared to control. These findings confirm previous locomotory analysis in zebrafish which revealed that zebrafish embryo single pulse static BDE-47 exposure at higher exposure concentrations (5–100 ppm) does not affect zebrafish locomotion at 4 and 6 dpf (Zhao et al., 2014). Together, the organismal level metabolic consequences of BDE-47 exposure affect similar endpoints as observed for PS exposure. An integrated overview of the organismal level phenotypic consequences of BDE-47 exposure is presented in Figure 10B.

### 4.3 Co-Exposure Enhances Organismal Level Metabolic Effects Observed for Individual PS and BDE-47 Exposures

Co-exposure of PS and BDE-47 increased zebrafish oxygen consumption-rate not only over control but also over BDE-47 and high PS. Similarly, feeding rate, a second endpoint observed to increase in response to PS and BDE-47 exposure alone further increased in PS + BDE-47 co-exposed zebrafish compared to BDE-47 and in the case of low PS exposed zebrafish, over PS alone. Thus, our data reveal that organismal level indices of energy balance are generally enhanced in co-exposures of PS and BDE-47. While the lack of internal BDE-47 and or PS sorption measurements precludes any conclusion regarding the importance of potential vector function of PS to enhance internal BDE-47 bioconcentration to mediate these effects, the additive and/or synergistic effects on early developing zebrafish energy metabolism, is further supported by a significant decrease of neutral lipid reserves in zebrafish co-exposed to high PS and BDE-47 compared to controls. This suggests that under high PS and BDE-47 co-exposure conditions, internal lipid energy reserves are increasingly mobilized to meet energetic demands. However, as in individual exposures, co-exposure did not affect morphometric indices and only mildly affected behaviour, which revealed a decrease in movement initiation events in high PS + BDE-47 compared to control, without however affecting distance, time spent on locomotion or speed. An integrated overview of the organismal level phenotypic consequences of enhanced organismal metabolic effects in response to PS and BDE-47 co-exposure is presented in Figure 10C. Given reports of mitochondrial disruption of both PS + BDE-47, further research should investigate whole zebrafish larval mitochondrial disruption of PS, BDE-47 and their combination in detail using adapted Seahorse assays (Souders et al., 2018).

### 4.5 High PS Exposure Attenuates BDE-47 Effects on Metabolic Gene Expression

To investigate potential molecular underpinnings indicative of metabolic disruption in PS, BDE-47 and PS + BDE-47 exposed zebrafish, we quantified the relative abundance of transcripts in whole larvae involved in lipid and glucose metabolism as well as peptides involved in (neuro)endocrine regulation of energy balance. Gene expression of *apoab,* but not *apoa1a,* was significantly induced by BDE-47, a response attenuated in co-exposure with high PS. The apolipoprotein genes *apoa1a* and *apoba* are comparatively well-characterized in early zebrafish development (Otis and Farber, 2016; Otis et al., 2019; Thierer et al., 2019; Templehof et al., 2021) and have been shown to be responsive to feeding status (Cruz-Garcia and Schlegel, 2014) and contaminants including BPA and PFOS as well as their replacement compounds BPS and F-53B (Sant et al., 2017; Wang W et al., 2018; Shi et al., 2019; Martínez et al., 2020). Gene expression of *apoba* is, similarly to *apoa1a* (Babin et al., 1997)*,* strongly induced in the yolk syncytial layer at 2 dpf (Thierer et al., 2019; Templehof et al., 2021). Following the transition to exogenous feeding, *aboba* becomes restricted to liver (Thierer et al., 2019; Templehof et al., 2021) while *apoa1a* is expressed in endosomes and lysosomes in the liver and intestine (Otis and Farber, 2016; Otis et al., 2019). Its functional relevance in early developmental lipid metabolism remains however unknown*,* as homozygous gene deletion did not elicit changes in lipid metabolism (Templehof et al., 2021). BDE-47 also induced *insb* but not *insa* expression in zebrafish larvae, an effect attenuated by PS co-exposure. *Insa* and *insb* transcript abundance in early zebrafish development from 0–6 dpf has been shown to strongly increase and decrease, respectively (Papasani et al., 2006). While *insa* expression is restricted to the developing pancreas where it strongly increases after hatching, *insb* expression has been localized to both head region and pancreas using *in situ* hybridization and is minimal after 2 dpf, suggesting non-metabolic functions (Papasani et al., 2006). This expression pattern has been confirmed in CrispR/Cas9 mutants which revealed that while morphologically unaffected, pancreatic insulin was completely absent in *insa* but not *insb* mutants (Mullapudi et al., 2019). Functionally, this translates to severe metabolic disturbances including hyperglycemia and reduced yolk lipid metabolization in *insa −/−* larvae, but not *insb −/−* larvae. However, the lack of metabolic roles appears to be linked to low expression of *insb* after 2 dpf, as its overexpression successfully lowers glucose levels, revealing its metabolic function (Mullapudi et al., 2019). Interestingly, co-exposure of BDE-47 and PS revealed a marginally significant trend for decrease of pancreatic expressed *insa*, suggesting that co-exposure of high PS and BDE-47 may result in pancreatic toxicity reported in response to PS using *in situ* staining (Brun et al., 2019) and POPs using a zebrafish GFP-based insulin reporter line (Sant et al., 2017). Given the reported glucoregulatory function of *insb*, BDE-47-dependent induction of *insb* may be reflective of alterations of glucose metabolism. This is further corroborated by the simultaneous induction of *pck1,* a transcriptional indicator of glucose metabolism in early developing zebrafish (Elo et al., 2007; Gut et al., 2013; Brun et al., 2019). Both PS nanoplastics and BDE-47 have been linked to pancreatic toxicity and dysregulation of glucose metabolism (Zhang et al., 2016; Brun et al., 2019) and future studies should investigate the possibility of individual or cumulative effects of PS and BDE-47 on pancreatic oxidative stress and function using reporter lines in detail. Expression of *pomca* and *pomcb* was induced by BDE-47 and attenuated by co-exposure with high PS. The *pomca* was the only paralogue transcript investigated that was also responsive to PS, as low PS exposure also resulted in a significant increase. Expression of *pomc* is restricted to the pituitary corticotrophs at 1 dpf and responsive to modulation (Hansen et al., 2003; Liu et al., 2003). Compared to mammals, the zebrafish *pomc* gene has both conserved and differential roles on organismal energy metabolism (Shi et al., 2020). While *pomca* knockout in zebrafish induces increased body weight as in mammalian taxa, this increase in body weight represents, in contrast to mammalian *Pomc* knockout models, not a feed-intake mediated obesity phenotype. Instead, *pomca* knockout weight gain in zebrafish is dependent on *pomca* encoded ACTH, hypocortisolism associated hyperandrogenism, and is accompanied by a reduction in oxygen consumption (Shi et al., 2020). This is in direct contrast to mammalian *Pomc* knockout models, whose obesity phenotype is mediated by the loss of central inhibition of the feeding circuitry. Together, the targeted gene expression analysis is indicative of widespread stimulation of BDE-47 on energy metabolism pathways in zebrafish larvae which include induction of transcripts relevant to lipid and glucose metabolism, as well as endocrine factors involved in the regulation of energy metabolism. Comparatively, PS nanoplastic exposure only elicits an increase in *pomca* in the low PS exposure group. However, in all co-exposure groups, PS nanoplastics dose-dependently attenuate gene expression induced by BDE-47 to control group levels suggesting interaction of both compounds at the gene expression level.

## 5 Conclusion

Our study reveals that nanoplastics and BDE-47 similarly affect organismal level metabolic phenotype in zebrafish larvae and that co-exposure exacerbates this effect. Under the experimental conditions tested, zebrafish larvae appear to compensate for contaminant-induced increases in energy expenditure by increasing food intake and depleting lipid reserves while reducing acute oxygen consumptions rates. These changes generally do not manifestation in global behavioural or morphometric effects. Given the sensitivity of early developmental periods to long-term metabolic effects (Martínez et al., 2020), future studies should investigate possible metabolic consequences along developmental trajectories. As mitochondrial modes of actions have been described for both PS and BDE-47, future studies investigating whether additive and/or synergistic effects of PS and BDE-47 occur at the level of mitochondrial respiration are warranted. While the interaction between PS and BDE-47 on energy metabolism is also evident at the transcript level, the directionality of changes is generally opposite to additive effects observed at the organismal level. Thus, caution is warranted when deducing functional interaction between PS nanoplastics and POPs based on (targeted) gene expression profiles. Indeed, the directionality (additivity/synergism or attenuation) of POP effects in zebrafish is not necessarily correlated with internal dosing, as microplastic absorption limited uptake of F-53B (a hydrophobic POP) but elicited both additive and attenuating effects on oxidative stress and immune function in zebrafish larvae (Wu et al., 2019; Yang et al., 2020).

Overall, this study clearly demonstrates cumulative effects of emerging nanoplastics compounds and persistent legacy contaminants on organismal energy balance in early development in zebrafish, a model relevant to both eco- and human toxicology. These findings thus provide novel mechanistic insight of cumulative metabolism-disrupting effects and raise concerns regarding possible impacts on aquatic wildlife and developmental origins of human metabolic disease.

## Data Availability

The raw data supporting the conclusion of this article will be made available by the authors, without undue reservation.

## Supplementary Material

Supplementary Figure 1 Fluorescence microscopy-based investigation of tissue distribution of fluorescently labelled PS nanoplastic particles in intestine, gill and liver of control. **(A-C)** and 25 ppm PS-exposed **(D-F)** adult zebrafish.
