# Supplementary material for: Metabolic Consequences of Developmental Exposure to Polystyrene Nanoplastics, the Flame Retardant BDE-47 and Their Combination in Zebrafish
Source: Front Pharmacol. 2022 Feb 16;13:822111. doi: 10.3389/fphar.2022.822111 (PMC8888882; doi:10.3389/fphar.2022.822111)
Supplement: Supplementary file 1 [file Table1.DOCX]

| Gene | NCBI Genbank ID | FW primer sequence | RV primer sequence | Tm (°C) |
| --- | --- | --- | --- | --- |
| *apoa1a* | NM_131128.1 | GAAGGCCTTCGAGTCCAACA | TCTGTGCCGAATGTGGTCCTC | 55 |
| *apoba* | XM_689735.9 | AGCTGAAGAACGCACTCTCC | GAACTTCAGGGCCGCATCTA | 57 |
| *insa* | NM_131056.1 | TAAGCACTAACCCAGGCACA | GATTTAGGAGGAAGGAAACC | 59 |
| *insb* | NM_001039064.1 | ACTCTTCACAGACTCTGCTC | ACAGATGCTGGGATGGAGAA | 59 |
| *pck* | NM_214751.1 | GCACGGAGTGTTTGTAGGG | GGTCTCGGTTCAGTTCACG | 56 |
| *pomca* | NM_181438.3 | GCCCCTGAACAGATAGAGCC | CTCGTTATTTGCCAGCTCGC | 54 |
| *pomcb* | NM_001083051.1 | TCCATCGAGCTCCAAAACCC | ACATTTTACGGTCTGCGT | 54 |
