# Supplementary figures and images for: Metabolic Consequences of Developmental Exposure to Polystyrene Nanoplastics, the Flame Retardant BDE-47 and Their Combination in Zebrafish
Source: Front Pharmacol. 2022 Feb 16;13:822111. doi: 10.3389/fphar.2022.822111 (PMC8888882; doi:10.3389/fphar.2022.822111)

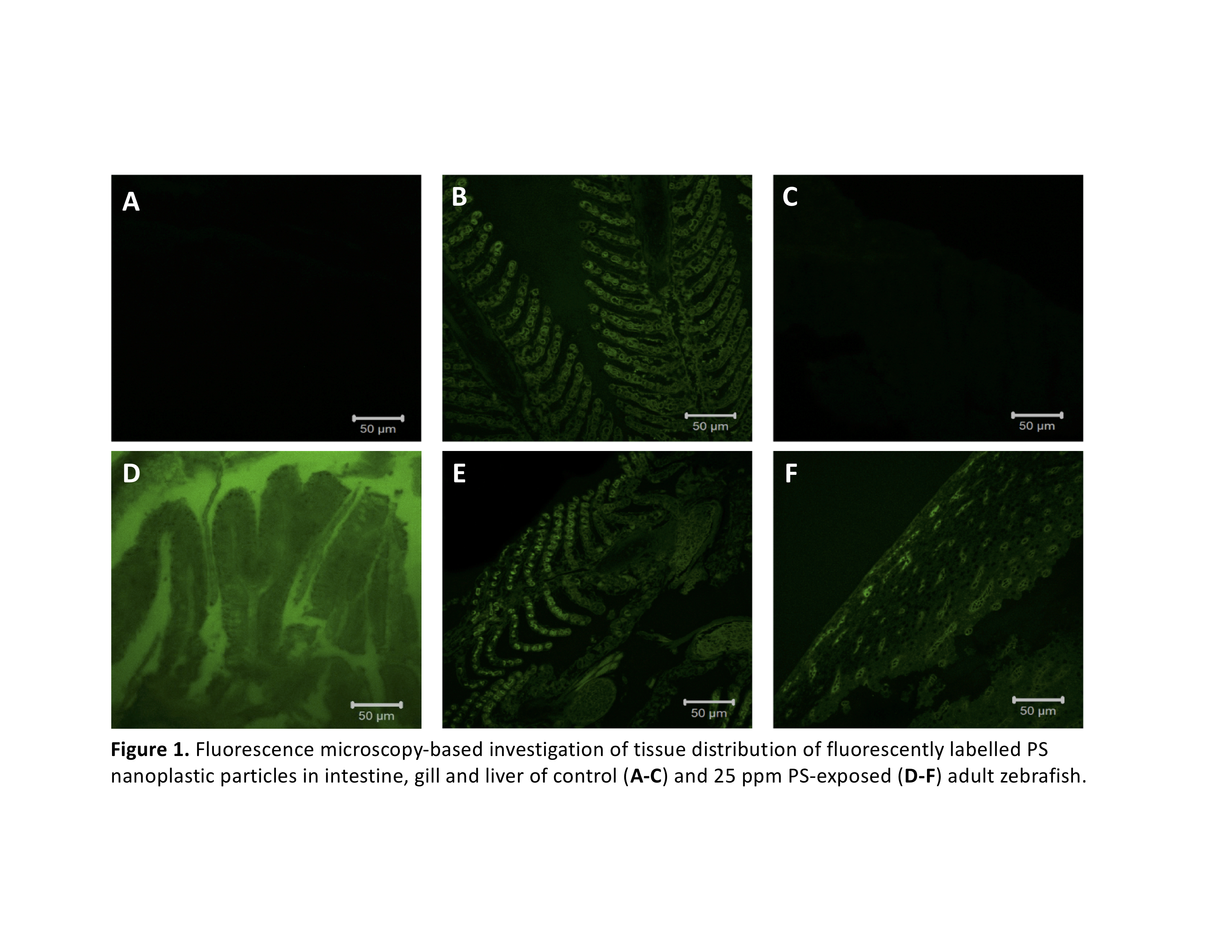

Supplement: Supplementary file 2 [file Image1.jpeg]
