# Supplementary material for: Metabolic Consequences of Developmental Exposure to Polystyrene Nanoplastics, the Flame Retardant BDE-47 and Their Combination in Zebrafish
Source: Front Pharmacol. 2022 Feb 16;13:822111. doi: 10.3389/fphar.2022.822111 (PMC8888882; doi:10.3389/fphar.2022.822111)
Supplement: Supplementary file 3 [file Table2.DOCX]

| Locomotion endpoint | Baseline | Dark | Light |
| --- | --- | --- | --- |
| Total movement counts | **df=5 F=2.641 p=0.024**  *η2p=0.47 Power=0.80* | **df=5 F=2.509 p=0.031**  *η2p=0.44 Power=0.78* | df=5 F=2.246 p=0.050  *η2p=0.40 Power=0.73* |
| Total movement duration | df=5 F=0.896 p=0.485  *η2p=0.16 Power=0.32* | df=5 F=0.765 p=0.575  *η2p=0.14 Power=0.27* | df=5 F=1.828 p=0.108  *η2p=0.33 Power=0.62* |
| Total movement distance | **df=5 F=2.509 p=0.031**  *η2p=0.44 Power=0.78* | df=5 F=1.747 p=0.124  *η2p=0.31 Power=0.60* | df=5 F=0.461 p=0.805  *η2p=0.08 Power=0.17* |
| Total movement speed | **df=5 F=3.216 p=0.008**  *η2p=0.57 Power=0.89* | **df=5 F=9.623 p=0.001**  *η2p=0.15 Power=1.00* | df=5 F=1.090 p=0.366  *η2p=0.20 Power=0.39* |
| Short movement counts | **df=5 F=2.898 p=0.014**  *η2p=0.05 Power=0.84* | **df=5 F=3.479 p=0.005**  *η2p=0.06 Power=0.91* | **df=5 F=2.465 p=0.044**  *η2p=0.04 Power=0.77* |
| Short movement duration | **df=5 F=3.161 p=0.009**  *η2p=0.06 Power=0.88* | **df=5 F=13.41 p=0.001**  *η2p=0.20 Power=1.00* | **df=5 F=6.236 p=0.001**  *η2p=0.10 Power=1.00* |
| Short movement distance | **df=5 F=4.411 p=0.001**  *η2p=0.08 Power=0.97* | **df=5 F=14.39 p=0.001**  *η2p=0.21 Power=1.00* | **df=5 F=7.163 p=0.001**  *η2p=0.12 Power=1.00* |
| Short movement speed | **df=5 F=6.876 p=0.001**  *η2p=0.12 Power=1.00* | **df=5 F=5.081 p=0.001**  *η2p=0.09 Power=0.98* | **df=5 F=4.081 p=0.001**  *η2p=0.07 Power=0.95* |
| Long movement counts | **df=5 F=2.259 p=0.049**  *η2p=0.04 Power=0.73* | df=5 F=1.727 p=0.129  *η2p=0.03 Power=0.59* | df=5 F=2.090 p=0.067  *η2p=0.04 Power=0.69* |
| Long movement duration | **df=5 F=2.270 p=0.048**  *η2p=0.04 Power=0.73* | **df=5 F=4.721 p=0.001**  *η2p=0.08 Power=0.98* | df=5 F=0.996 p=0.421  *η2p=0.02 Power=0.35* |
| Long movement distance | **df=5 F=4.929 p=0.001**  *η2p=0.08 Power=0.98* | **df=5 F=6.123 p=0.001**  *η2p=0.10 Power=1.00* | df=5 F=1.495 p=0.191  *η2p=0.03 Power=0.52* |
| Long movement speed | **df=5 F=3.654 p=0.003**  *η2p=0.07 Power=0.93* | df=5 F=1.398 p=0.255  *η2p=0.03 Power=0.49* | df=5 F=0.709 p=0.617  *η2p=0.01 Power=0.26* |
