# Supplementary material for: Metabolic Consequences of Developmental Exposure to Polystyrene Nanoplastics, the Flame Retardant BDE-47 and Their Combination in Zebrafish
Source: Front Pharmacol. 2022 Feb 16;13:822111. doi: 10.3389/fphar.2022.822111 (PMC8888882; doi:10.3389/fphar.2022.822111)
Supplement: Supplementary file 4 [file Table3.DOCX]

| Locomotion endpoint | Baseline | Dark | Light |
| --- | --- | --- | --- |
| Total movement counts | **df=2 F=5.013 p=0.007** | df=2 F=0.870 p=0.420 | df=2 F=0.637 p=0.529 |
| Total movement duration | **df=2 F=4.462 p=0.012** | df=2 F=0.120 p=0.887 | df=2 F=0.61 p=0.941 |
| Total movement distance | **df=2 F=10.10 p=0.001** | **df=2 F=6.358 p=0.002** | **df=2 F=5.871 p=0.003** |
| Total movement speed | **df=2 F=6.287 p=0.002** | **df=2 F=22.63 p=0.001** | df=2 F=1.169 p=0.312 |
| Short movement counts | **df=2 F=4.578 p=0.011** | df=2 F=1.013 p=0.365 | df=2 F=0.999 p=0.370 |
| Short movement duration | **df=2 F=1.763 p=0.174** | **df=2 F=10.63 p=0.001** | **df=2 F=8.238 p=0.001** |
| Short movement distance | df=2 F=2.620 p=0.075 | **df=2 F=12.105 p=0.001** | **df=2 F=9.279 p=0.001** |
| Short movement speed | **df=2 F=4.058 p=0.018** | df=2 F=2.596 p=0.077 | **df=2 F=3.525 p=0.031** |
| Long movement counts | **df=2 F=5.141 p=0.006** | df=2 F=1.100 p=0.335 | df=2 F=0.618 p=0.540 |
| Long movement duration | **df=2 F=7.918 p=0.001** | **df=2 F=7.074 p=0.001** | **df=2 F=8.923 p=0.001** |
| Long movement distance | **df=2 F=12.283 p=0.001** | **df=2 F=14.594 p=0.001** | **df=2 F=11.56 p=0.001** |
| Long movement speed | **df=2 F=13.72 p=0.001** | **df=2 F=13.79 p=0.001** | df=2 F=1.959 p=0.143 |
